# Molecular mechanism of cholesterol-dependent membrane fusion in SARS-CoV-2 entry

**DOI:** 10.1038/s41392-026-02573-z

**Published:** 2026-02-17

**Authors:** Wei Li, Mengdan Wu, Shirong Feng, Jiaming Su, Qian Niu, Weijun Lin, Jiaqi Fan, Lele Cui, Yijuan Xiang, Hao Li, Kaiyu Li, Zhaoming Su, Guangwen Lu, Ying Lai

**Affiliations:** 1 National Clinical Research Center for Geriatrics, State Key Laboratory of Biotherapy, West China Hospital, Sichuan University, Chengdu, Sichuan China; 2 Cancer Institute (Key Laboratory of Cancer Prevention and Intervention, China National Ministry of Education), The Second Affiliated Hospital, Zhejiang University School of Medicine, Hangzhou, Zhejiang China; 3 Department of Laboratory Medicine/Clinical Laboratory Medicine Research Center, West China Hospital, Sichuan University, Chengdu, China; 4 West China Hospital Emergency Department, State Key Laboratory of Biotherapy, West China Hospital, Sichuan University, Chengdu, Sichuan China

**Keywords:** Biophysics, Cell biology, Microbiology

## Abstract

Enveloped virus invasion relies on spike glycoprotein-mediated membrane fusion. Cholesterol that serves crucial roles in modulating protein conformations and membrane properties, plays an essential role in the severe acute respiratory syndrome coronavirus 2 (SARS-CoV-2) cell entry. However, the precise regulatory mechanism of cholesterol in SARS-CoV-2 fusion remains unknown. Here, using an in vitro vesicle-vesicle content mixing assay, we demonstrated that the addition of cholesterol enhanced SARS-CoV-2 spike-mediated vesicle-vesicle fusion, with this enhancement being dependent on the C-terminal cytoplasmic domain of spike. Further single-vesicle analyses demonstrate this enhancement primarily stems from increased docking probability, with cholesterol exerting mild effect on fusion probabilities. In the cell-based membrane fusion assay, cholesterol depletion from spike containing membrane significantly reduces syncytia formation and SARS-CoV-2 pseudovirus infection, indicating its modulatory role in this process. Using structured illumination microscopy (SIM) based super-resolution imaging and single-molecule photobleaching microscopy, we demonstrated that spike proteins tended to form into an oligomeric cluster in the presence of cholesterol, likely through the interaction between cholesterol and palmitoylated cysteine rich region (CRR) in the C-terminus of spike. Last, substitution of residues of CRR with alanine in the C-terminus of spike abolished both the cholesterol-induced spike clustering and the cholesterol-dependent enhancement of vesicle docking. Taken together, our results suggest that cholesterol may induce the oligomerization of spike through specific interactions with its CRR, with this structural clustering critically mediating viral docking to host cell membranes, thereby promoting the subsequent membrane fusion and viral entry processes.

## Introduction

SARS-CoV-2, an enveloped positive-sense single-stranded RNA virus,^1^ is the third zoonotic coronavirus that emerged after severe acute respiratory syndrome-associated coronavirus (SARS-CoV, 79.5% sequence identity) and Middle East respiratory syndrome coronavirus (MERS-CoV, 50% sequence identity), which exhibits high infectivity and mortality.^2^ The invasion mechanism of coronavirus is conserved, represented by SARS-CoV-2, which relies on the surface glycoprotein spike to recognize the angiotensin-converting enzyme 2 (ACE2) receptor on the host cell plasma membrane,^3–5^ further initiating viral membrane to fuse with the host cell membrane or uptake through endocytic pathway.^6,7^

The SARS-CoV-2 spike glycoprotein is a type I membrane protein,^6^ comprising an S1 receptor recognition subunit and an S2 fusion subunit connected by two proteolytic cleavage sites (S1/S2 and S2’) that are required for fusion conformational rearrangements.^8^ In the pre-fusion state, three receptor-binding S1 heads are positioned atop a trimeric membrane-fusion S2 stalk,^6^ and an ‘up’ conformation of receptor-binding domain (RBD) in a trimerized S1 subunits binds to the receptor ACE2. Subsequently, the S2’ site is cleaved by the host surface transmembrane protease serine 2 (TMPRSS2) or cathepsin L in the endosomal compartment,^6,9^ followed by shedding of the S1 subunit from the S2 subunit, and a cascade refolding event to form a six-helix bundle (6-HB) from the helical regions of HR1 and HR2 in the S2 subunit. During the formation of a stable rigid post-fusion structure, the insertion of the fusion peptide into the target membrane and the dramatic conformational change of S2 subunit propels membrane fusion between the virus and the host cell membrane.^10^ This multi-step process is highly sensitive to and regulated by the local membrane environment, particularly its lipid composition.

It was reported that the cytosolic tail of SARS-CoV-2 spike could interact with coat protein to regulate its transport pathway, membrane localization,^11^ and viral infectivity.^12–14^ The cytoplasmic domain sequences of SARS-CoV and SARS-CoV-2 are highly conserved, with the exception of a unique cysteine residue insertion in SARS-CoV-2, which results in differential palmitoylation patterns mediated by zinc finger DHHC domain-containing palmitoyltransferases through their enzymatic cascade.^12^ Palmitoylated modification enhances protein hydrophobicity, thereby promoting the interaction with lipid rafts (glycosphingolipid/cholesterol-rich microdomains) and membrane adhesion.^15^ Treatment with 25-hydroxycholesterol reduces the plasma membrane cholesterol levels and promotes lipid droplet formation, which in turn impairs SARS-CoV-2 entry into host cells.^16^ In the SARS-CoV-2 pseudovirus entry model, removal of cholesterol from the viral membrane, as opposed to the host membrane, leads to inhibition of viral infection.^17^ In vitro, the S1 subunit of SARS-CoV-2 spike was reported to bind to cholesterol and possibly to high-density lipoprotein (HDL) components to facilitate viral uptake.^18^ Early work on viral infection showed that SARS-CoV-2 achieves membrane fusion by cholesterol altering the oligomeric state of the fusion peptide.^19,20^ Moreover, cholesterol, alongside ceramide, enhances membrane fusion mediated by the SARS-CoV-2 spike fusion peptide in liposome–liposome and cell–cell fusion assays.^21^ While existing evidence underscores the importance of cholesterol as a key modulator of spike-mediated fusion, the precise mechanisms by which it regulates full-length spike protein conformational dynamics, receptor binding, and the subsequent fusion steps remain incompletely understood. Furthermore, the potential role of cytoplasmic domain of the spike protein, which interacts with cellular machinery and undergoes palmitoylation, in sensing membrane cholesterol during the entry process remains virtually unexplored. A major technical challenge has been the difficulty of disentangling the role of cholesterol in distinct stages of membrane fusion, within a physiologically relevant context that preserves native spike protein topology and mobility.

To address these specific questions, we developed a physiologically relevant reconstitution system. In this work, the purified engineered spike protein was reconstituted onto a group of liposomes to mimic the viral membrane, while the soluble ecto-domain of ACE2 protein was reconstituted onto another set of liposomes to mimic the host cell membrane. This optimized vesicle-vesicle fusion system, combined with single-vesicle imaging techniques, allowed us to decouple the substeps of membrane fusion, and to precisely control the lipid composition of each membrane population. Utilizing this optimized vesicle-vesicle fusion system, we determined that cholesterol on the spike-vesicle membrane had an enhancement effect on the efficiency of spike-mediated vesicle fusion. Importantly, we have consistently observed this cholesterol-dependent enhancement of fusion not only in our reconstituted system but also in cell–cell fusion and pseudotyped viral entry assays. Additionally, we found that cholesterol specifically promotes the spike-mediated vesicle docking via the C-terminal cytoplasmic region of spike in the single-vesicle fusion assay. Moreover, the CRR in the cytoplasmic domain of spike protein is prone to form high-density and large-diameter clusters via colocalizing with cholesterol in membrane. Finally, the cluster of spike mediated by the interaction between spike CRR and cholesterol is critical for spike-mediated viral membrane docking to host cell membrane, indicating a potential target to inhibit coronavirus infection like SARS-CoV-2 transmission.

## Results

### Cholesterol facilitates spike-mediated membrane fusion in reconstituted vesicle-vesicle fusion system

**Fig. 1:**
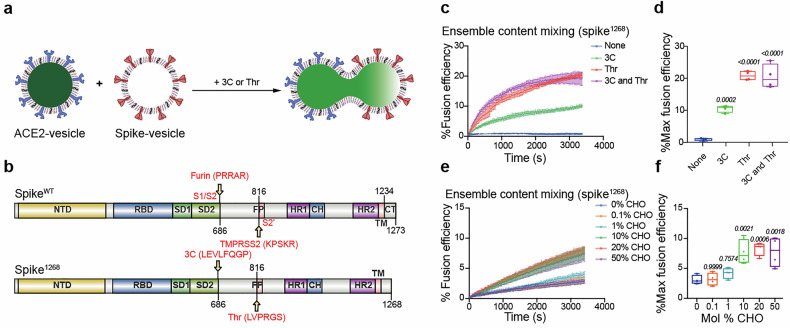
Cholesterol-dependent membrane fusion mediated by spike protein in a reconstituted vesicle-vesicle content-mixing system. **a** Schematic diagram of the ensemble content-mixing assay between spike-vesicles and ACE2-vesicles. Proteolytic treatment was performed using 10 U 3C or 5 U Thr protease, and the fluorescence of dequenching signal of SRB was monitored. **b** Domain schematic of spike^1268^ in which functional domains and cleavage sites are annotated. In the mutants, the furin cleavage site (PRRAR, S1/S2) was mutated into the 3C cleavage site (LEVLFQGP), while the TMPRSS2 cleavage site (KPSKR, S2’) was mutated into the Thr cleavage site (LVPRGS). Meanwhile, 5 amino acids from the C-terminal cytoplasmic domain of spike^WT^ were deleted to obtain spike^1268^. NTD, N-terminal domain; RBD, receptor-binding domain; SD1, subdomain 1; SD2, subdomain 2; FP, fusion peptide; HR1, heptad repeat 1; CH, central helix; HR2, heptad repeat 2. **c** The ensemble content mixing assay of the spike^1268^-vesicles with ACE2-vesicles. The fluorescence change of SRB was normalized with respect to the maximum fluorescence intensity obtained by adding 0.1% Triton X-100. Traces represent the mean ± SEM from *N* = 4 independent replicates. **d** Box plots and data points show the maximum fusion efficiency corresponding to panel (**c**). **e** The ensemble content mixing of cholesterol titration experiment with spike^1268^-vesicles (0–50 mol% cholesterol) fused with ACE2-vesicles (20 mol% cholesterol). Traces represent the mean ± SEM from *N* = 4 independent replicates. CHO, cholesterol. **f** Box plots and data points show the maximum fusion efficiency corresponding to panel (**e**). In panel (**d**), statistical analysis was performed using one-way ANOVA followed by Tukey’s multiple comparisons test. In panel (**f**), statistical analysis was performed using one-way ANOVA followed by Dunnett’s multiple comparisons test

We previously developed a cell-vesicle fusion system that successfully mimics the process of spike-mediated viral invasion,^22^ but the spike-expressing cell could not reflect the physiological membrane curvature of viral membrane and non-specific uptake of vesicles by the host cells may lead to elevated background signals. To address these issues, we reconstituted purified spike protein onto one set of liposomes (i.e., spike-vesicle) to mimic virus membrane, while the extracellular domain of ACE2 was reconstituted onto a separate set of liposomes containing sulforhodamine B (SRB) via 6×histidine_Ni-NTA interaction (i.e., ACE2-vesicle) to mimic host cell membrane (Fig. 1a). To generate a stable and highly expressed spike protein for our studies, we created the spike^1268^ construct. As our previous work, the endogenous S1/S2 and S2’ cleavage sites of spike were replaced with prescission (3C) and thrombin (Thr) cleavage sites to prevent auto-cleavage during expression, and the residues in C-terminal part (aa^1269^-aa^1273^) of spike were deleted to increase its expression on the cell surface (Fig. 1b). The outward orientation of spike on vesicles was confirmed by pepsin digestion (Supplementary Fig. 1a). The reconstituted proteins of spike^1268^-vesicles and ACE2-vesicles were examined via SDS-PAGE (Supplementary Fig. 1b), while their morphology was characterized by cryo-electron microscopy (cryo-EM) with an average diameter of 51.4 nm and 54.1 nm, respectively (Supplementary Fig. 1c, d). The lipid compositions of both spike- and ACE2-containing vesicles were designed to mimic key features of viral membrane and plasma membranes,^23–26^ with adjustments to accommodate protein reconstitution while maintaining physiological relevance. Membrane fusion occurred upon mixing the spike-vesicle and ACE2-vesicle (Fig. 1c). Content mixing between the spike-vesicle and ACE2-vesicle was indicated by the rise of the dequenching signal of SRB in post-fusion.

Consistently, when spike^1268^-vesicle and ACE2-vesicle were mixed, membrane fusion occurred in the presence of Thr or 3C protease (Fig. 1c). As a control, little fusion was observed in the absence of protease (Fig. 1d). The fusion efficiency of content mixing induced by the cleavage of S2’ site was significantly higher than that induced by the cleavage of the S1/S2 site (Fig. 1d), suggesting that spike-mediated membrane fusion rely on the exposure of fusion peptide via cleavage at the S2’ site. The addition of both 3C and Thr proteases did not result in further increase in fusion efficiency compared to the addition of Thr protease alone (Fig. 1d). In all subsequent experiments, 5 U Thr protease was applied to induce cleavage at the S2’ site unless otherwise specified.

### Cholesterol enhances the spike^1268^-mediated vesicle docking

Cholesterol has been documented to impact membrane fusion via altering lipid fluidic properties, or regulating the conformation of fusogenic protein.^27^ In the ensemble vesicle-vesicle content mixing assay, we observed a significant increase in the fusion probability between spike^1268^-vesicle and ACE2-vesicle when high concentration (≥10%) of cholesterol was included on the spike^1268^-vesicle (Fig. 1e, f). In contrast, alterations of cholesterol concentration in ACE2-vesicle demonstrated mild impact on bulk vesicle-vesicle fusion efficiency, with a significant inhibitory effect observed at the high cholesterol level of 50 mol%, which probably result from an effect of lipid fluidity by overloaded cholesterol (Supplementary Fig. 2a, b). Note that, we also performed a bulk fusion assay between ACE2-expressing cells and spike-vesicle with different cholesterol concentrations. These cell-vesicle fusion experiments demonstrated a cholesterol-dependent enhancement of spike-mediated fusion efficiency across the physiological concentration gradient (Supplementary Fig. 3a, b).

We also examined the effect of lipid composition on the ensemble content mixing (Supplementary Fig. 4a, b). The results revealed that the absence of 7.5% phosphatidylinositol (PI) did not affect the efficiency of content mixing (Supplementary Fig. 4). Although the replacement of 20% 1,2-dipalmitoyl-sn-glycero-3-phosphoethanolamine (DOPE, PE) or 15% 1,2-dioleoyl-sn-glycero-3-phospho-L-serine (DOPS, PS) by 1-palmitoyl-2-oleoyl-sn-glycero-3-phosphocholine (POPC, PC) led to reduction in the efficiency of content mixing (Supplementary Fig. 4a, b), we doubt that it might result from liposome abnormal aggregation or deformation induced by the depletion of key phospholipids like PE or PS (Supplementary Fig. 5).

**Fig. 2:**
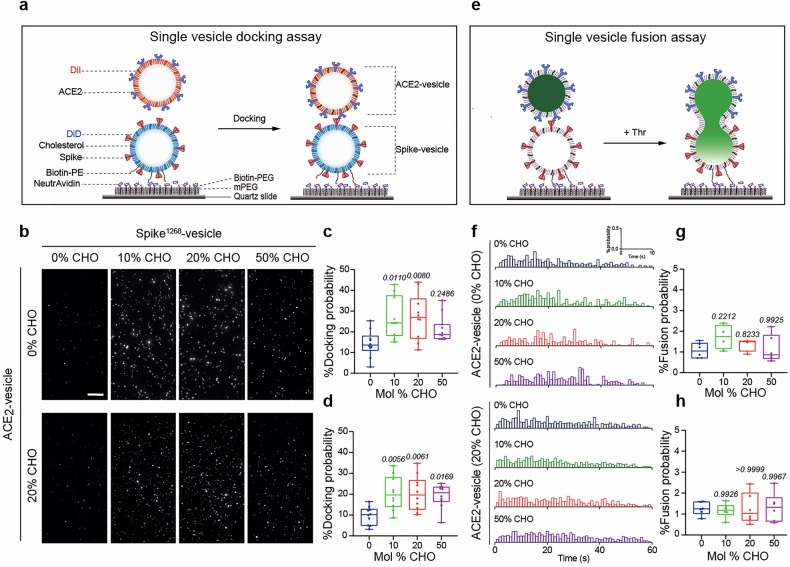
Cholesterol specifically enhances spike^1268^-mediated vesicle docking without altering fusion kinetics. **a** Schematic of the single-vesicle lipid mixing assay for spike-mediated vesicle docking. **b** Representative fields of view of the fluorescent spots of ACE2-vesicles docking to the spike-vesicles. The fluorescent spots correspond to ACE2-vesicles docking with spike-vesicles which were immobilized on the passivated surface. The representative fields of view are docking events of ACE2-vesicles (0 or 20 mol% cholesterol) with spike^1268^-vesicles with cholesterol ranging from 0 to 50 mol%. Scale bars, 10 μm. Scale bar applies to all images in this panel. **c** Box plots and data points show the docking probability for ACE2-vesicles (0 mol% cholesterol) corresponding to panel (**b**) from *N* = 10 independent replicates. **d** Box plots and data points show the docking probability for ACE2-vesicles (20 mol% cholesterol) corresponding to panel (**b**) from *N* = 10 independent replicates. **e** Schematic of the single-vesicle content mixing assay for spike-mediated vesicle fusion. **f** Corresponding fusion histograms of ACE2-vesicles (0 mol% cholesterol, upper; 20 mol% cholesterol, lower) with spike^1268^-vesicles with cholesterol ranging from 0 mol% to 50 mol%. **g** Box plots and data points show the fusion probability for ACE2-vesicles (0 mol% cholesterol) corresponding to panel (**f**) from *N* ≥ 4 independent replicates. **h** Box plots and data points show the fusion probability for ACE2-vesicles (20 mol% cholesterol) corresponding to panel (**f**) from *N* = 6 independent replicates. CHO, cholesterol. In panels (**c**), (**d**), (**g**) and (**h**), statistical analysis was performed using one-way ANOVA followed by Tukey’s multiple comparisons test

We next utilized a single-vesicle docking assay to investigate the effect of cholesterol on vesicle docking between spike-vesicle and ACE2-vesicle (Fig. 2a). In this assay, a fluorescent pair of DiD and DiI was included in spike-vesicle and ACE2-vesicle, respectively. Spike-vesicles were tethered onto a PEGylated quartz surface via a specific interaction between neutravidin and biotin-conjugated PE (biotin-PE). Docked ACE2-vesicles were analyzed by counting colocalized spike-vesicle and ACE2-vesicle (Fig. 2a). By incorporating cholesterol into the spike-vesicle, we observed a significant increase in the docking probability between spike^1268^-vesicle and ACE2-vesicle (Fig. 2b–d). However, the percentage of high-FRET population of single-vesicle lipid mixing between spike^1268^-vesicle and ACE2-vesicle only exhibited a mild change in the presence of Thr protease (Supplementary Fig. 6a–c). Note that lipid mixing-based assays do not reflect the full fusion, i.e., the release of content, despite their success,^28^ as the high FRET fraction of lipid mixing may result from the exchange of lipid molecules between closely contacted membranes. We therefore employed a single-vesicle content mixing assay (Fig. 2e). In this assay, unlabeled spike-vesicles with biotinylated lipids were tethered on a passivated surface, while ACE2-vesicle encapsulating self-quenched SRB were loaded. Upon removal of unbound ACE2-vesicles and addition of Thr protease, fusion events occurred, resulting in an increase in the fluorescence intensity of SRB. We observed that the fusion probability of spike-mediated content mixing was approximately 1 ~ 2%, relatively low compared to that mediated by other fusogens like SNARE proteins,^29^ indicating the viral infection is a slow process compared to cell secretion (Fig. 2f–h). Consistently, the fusion probability remained unchanged as the molar fraction of cholesterol on the spike^1268^-vesicle membrane ranged from 0 mol% to 50 mol%, regardless of the presence of cholesterol in ACE2-vesicle (Fig. 2g, h). Consistent with the ensemble fusion assays, elevating cholesterol in ACE2-vesicles did not enhance either the docking probability (Supplementary Fig. 7a, b) or fusion probability (Supplementary Fig. 7c, d) to spike^1268^-vesicles, except that it revealed a discernible inhibitory effect only at 50 mol% cholesterol (Supplementary Fig. 7c, d). Thus, cholesterol may regulate spike^1268^-mediated vesicle docking rather than vesicle fusion, explaining the particular stimulatory effect of cholesterol observed in the bulk content mixing assay (Fig. 1e, f).

### Cholesterol is required for spike^1268^-mediated cell-based membrane fusion

**Fig. 3:**
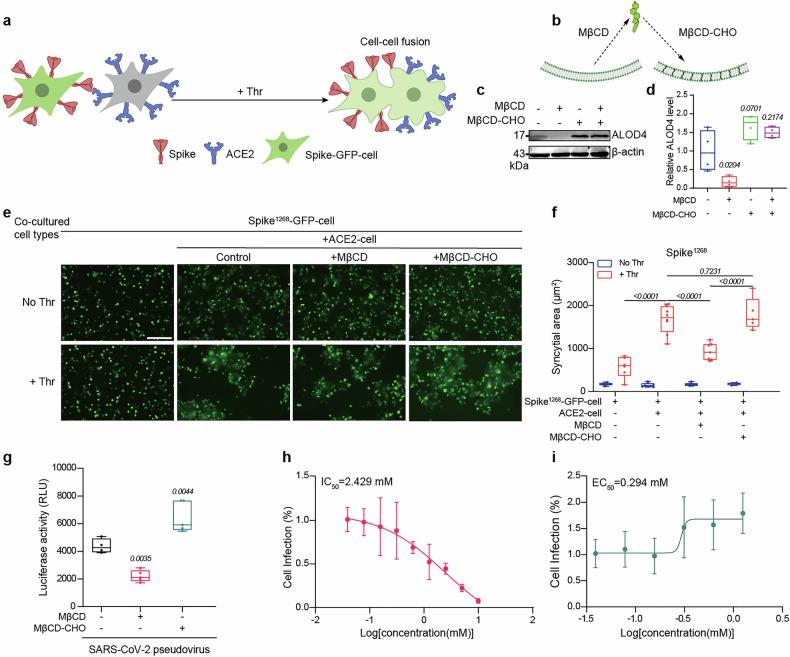
Cholesterol potentiates spike^1268^-mediated viral infection. **a** Schematic diagram of the cell–cell fusion assay for ACE2-cell and spike-EGFP-cell pretreated with MβCD or MβCD-conjugated cholesterol. **b** Schematic diagrams of cholesterol treatments on cell membranes with MβCD or MβCD-CHO. **c** Western blot analysis of cholesterol content in the plasma membrane by ALOD4. **d** Box plots and data points show the cholesterol content on cell membrane treated with MβCD or MβCD-CHO corresponding to panel (**c**). **e** Syncytia formation induced by spike^1268^. Spike^1268^-EGFP-cell with different pre-treatments and ACE2-cell were co-cultured at a 2:1 ratio with or without 5 U Thr protease. Scale bars, 200 μm. Scale bar applies to all images in this panel. **f** Box plots and data points show the syncytial area in cell–cell fusion corresponding to panel (**e**) from *N* = 5 independent replicates. The area of syncytia was calculated based on the fused cells with weak fluorescence intensity of EGFP and was at least twice as large as the unfused cells. At least five fields were randomly selected in each well. **g** Infection of SARS-CoV-2 pseudovirus into hACE2-expressing 293T cells under different treatments of cholesterol on cell membrane (*N* ≥ 4). Luciferase activity was measured to quantify the infection efficiency. **h** A dose-dependent inhibitory effect on pseudovirus infection by depletion of cholesterol. SARS-CoV-2 pseudovirus was pre-treated with increasing concentrations of MβCD (0.039–10 mM). Dose dependent-response curve fitting with four-parameter logistic function revealed an IC_50_ value of 2.429 mM. **i** A dose-dependent enhancement effect on pseudovirus infection by the replenishment of cholesterol. SARS-CoV-2 pseudovirus was pre-treated with increasing concentrations of MβCD-CHO (0.039–1.25 mM), Dose dependent-response curve fitting with four-parameter logistic function revealed an EC_50_ of 0.294 mM. In panels (**d**), (**f**), (**g**), statistical analysis was performed using one-way (**d**, **g**) or two-way (**f**) ANOVA followed by Tukey’s multiple comparisons test

We next investigated the role of cholesterol in a cell–cell fusion assay by modulating cholesterol levels on the cell membrane. In this fusion assay, one group of HEK293T cells was co-transfected with the plasmids encoding EGFP and spike^1268^ (i.e., spike^1268^-EGFP-cell), while another group of HEK293T cells was transfected with the plasmid encoding human ACE2 (i.e., ACE2-cell) (Fig. 3a). Upon coculturing the two groups of cells, syncytia formation was visualized based on the expanded area of EGFP fluorescent reporter if fusion occurred. The efficiency of cell–cell fusion was quantified by summarizing the area of syncytia in each image.

In this cell–cell fusion assay, methyl-β-cyclodextrin (MβCD) was utilized to deplete cholesterol from the spike-cell membrane via forming soluble inclusion complexes, while its cyclodextrin complex (MβCD-conjugated cholesterol, MβCD-CHO) was used to replenish cholesterol in the cell membrane^16^ (Fig. 3b). Anthrolysin O domain 4 (ALOD4), a bacterial toxin was employed to monitor the concentration of cholesterol in spike-cell membrane after pretreatment^30^ (Fig. 3c, d and Supplementary Fig. 8), and we found that the relative amount of cholesterol can be maintained during 48 h of incubation for syncytia formation (Supplementary Fig. 8). We discovered that the syncytia formation was drastically inhibited by the treatment of spike^1268^-EGFP-cell with MβCD, and this suppression effect could be rescued by the supplement of MβCD-CHO (Fig. 3e, f). Compared to the non-treated spike^1268^-EGFP-cell, there was no further enhancement in cell–cell fusion with the additional replenishment of cholesterol (Fig. 3e, f). As a control, we did not observe any effect on the expression of spike protein on cell membrane, and cell viability under different pretreatments of cholesterol by MβCD or MβCD-CHO (Supplementary Fig. 9a–h). Note that, we also observed the consistent cholesterol-dependent regulatory trend with wild-type of spike^WT^ (i.e., spike^WT^-EGFP-cell) (Supplementary Fig. 10a–c). In summary, our results suggest that cholesterol is required for spike-mediated cell–cell fusion.

We next asked whether modulating the cholesterol level in SARS-CoV-2 virus affects its infectivity. We pre-treated SARS-CoV-2 pseudovirions with MβCD to deplete cholesterol or with MβCD-CHO to enrich cholesterol. Consistently, cholesterol depletion significantly impaired pseudovirus infection, while cholesterol replenishment enhanced it (Fig. 3g). Moreover, titration of MβCD yielded a dose-dependent inhibition of infection with a half-maximal inhibitory concentration (IC_50_) of 2.429 mM (Fig. 3h). Conversely, titration of MβCD-CHO produced a dose-dependent enhancement of infectivity with a half-maximal effective concentration (EC_50_) of 0.294 mM (Fig. 3i). However, modulating cholesterol levels in ACE2-expressing cells via MβCD or MβCD-CHO had no significant effect on the fusion efficiency with spike-vesicle in ensemble fusion assay (Supplementary Fig. 11a, b), syncytia formation (Supplementary Fig. 12a, b) or pseudovirus infection (Supplementary Fig. 12c). As a control, we did not observe any effect on the expression of ACE2 on cell membrane, and cell viability under different pretreatments of MβCD or MβCD-CHO (Supplementary Fig. 13a–c).

Taken together, our results from vesicle-vesicle fusion, cell–cell fusion, and pseudovirus infection assays provide convergent evidence that membrane cholesterol is an essential factor on viral membrane to facilitate SARS-CoV-2 spike-mediated viral entry.

### Cholesterol induced the cluster formation of spike^1268^

**Fig. 4:**
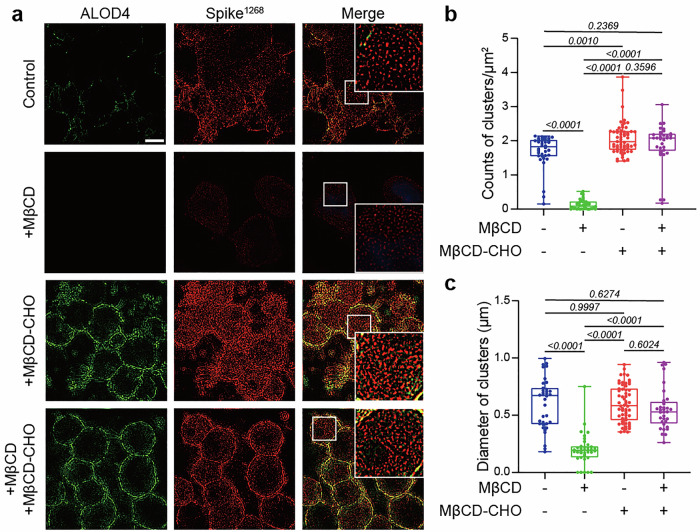
Cholesterol drives the cluster formation of spike^1268^ protein. **a** Reconstructed SIM images illustrating cholesterol-modulated spatial distribution of spike^1268^ clusters. Among the groups, the +MβCD + MβCD-CHO group involved pre-treating cells with MβCD to deplete membrane cholesterol, followed by the addition of MβCD-CHO to rescue the cell membrane cholesterol. Scale bars, 5μm. Scale bar applies to all images in this panel. **b** Box plots and data points show the counts of clusters per μm^2^ for the spike^1268^ protein corresponding to panel (**a**) from *N* ≥ 3 independent replicates. **c** Box plots and data points show the size of clusters (Feret’s diameter) corresponding to panel (**a**) from *N* ≥ 3 independent replicates. Cluster quantification and diameter measurements were performed using NIS-Elements software, with ≥20 randomly selected cells in each group. Statistical analysis was performed using one-way ANOVA followed by Tukey’s multiple comparisons test

To explore the mechanism of cholesterol-dependent viral docking process, we employed SIM to investigate the spatial distribution of spike on cell membranes. Cells pretreated with MβCD or MβCD-CHO were labeled by Cy3-conjugated ALOD4, which recognizes cholesterol specifically,^31^ while spike protein was labeled by APC-conjugated anti-spike antibody. SIM analysis demonstrated that spike^1268^ (Fig. 4a–c) and spike^WT1268^ (Supplementary Fig. 14a–c) proteins tended to form dense clusters on the cholesterol-containing cell membrane, while appearing more discrete on cholesterol-deficient cell membranes. And the size of the spike clusters in nanoscale domains on cholesterol-sufficient membrane was significantly larger than that on cholesterol-deficient cell membranes (Fig. 4a–c and Supplementary Fig. 14a–c). This increased protein accumulation likely underpins the enhanced spike-mediated viral docking process, highlighting the critical role of cholesterol in facilitating this interaction.

### Cholesterol regulates the oligomeric state of spike^1268^ clusters

**Fig. 5:**
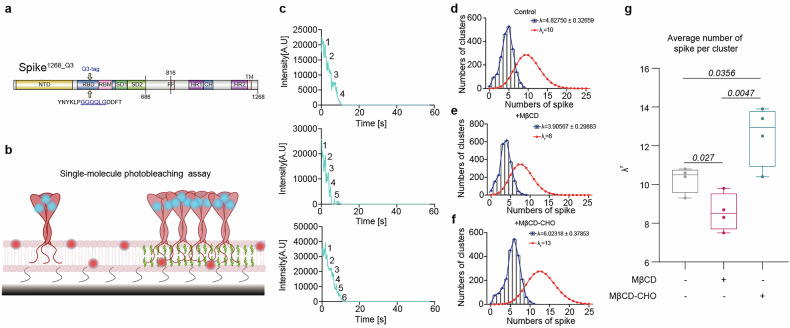
Cholesterol depletion disrupts the oligomerization of spike^1268^ in native plasma membranes. **a** Schematic diagram of spike^1268_Q3^, highlighting the N-terminal Q3 tag proximal to the receptor-binding motif (RBM). **b** Schematic of single-molecule photobleaching assay for spike^1268^ protein. **c** Representative time traces showing single-molecule stepwise photobleaching events with the fluorophore Cy5. Sequential fluorophore quenching reflects oligomeric states. **d** Distribution of the number of spike^1268^ monomers per cluster on control SLBs. Blue curves: labeled monomers (λ); red curves: total monomers (λ_r_, labeled + unlabeled). **e** Distribution of the number of spike^1268^ monomers per cluster on MβCD treated SLBs. **f** Distribution of the number of spike^1268^ monomers per cluster on MβCD-CHO treated SLBs. **g** Box plot and data points show the average number (λ_r_) of spike^1268^ per cluster on SLB (control, MβCD, MβCD-CHO), which is obtained from the calculated Poisson distribution of all bound spike^1268^ monomers (i.e., red curves in **d**–**f**). Statistical analysis was performed using t-test

To investigate cholesterol-dependent oligomerization dynamics of spike^1268^ at single-molecule level, we utilized a site-specific bioconjugation strategy via genetic insertion of a Q3 peptide tag (GQQQLG) at the N-terminus of spike^1268^ (designated spike^1268_Q3^) (Fig. 5a). The enzymatic labeling with Cy5 fluorophores was achieved via microbial transglutaminase (gpTGase)-mediated crosslinking (Fig. 5a).

Subsequent reconstitution of Cy5 labeled spike^1268_Q3^ onto supported lipid bilayers (SLBs) containing specified cholesterol (0 mol% vs 20 mol%) enabled quantitative analysis of oligomeric states through single-molecule photobleaching assay (Fig. 5a, b and Supplementary Fig. 15). Distinct photobleaching step-wise patterns corresponding to individual Cy5-labeled spike^1268_Q3^ were resolved (Supplementary Fig. 15a). To compensate for incomplete labeling efficiency, we implemented Poisson statistics correction (λr) to calculate total copy numbers per cluster, supporting a stochastic monomer incorporation mechanism during cluster formation (Supplementary Fig. 15b, c). The observed step-count distributions demonstrated strong agreement with Poisson models. The clusters in cholesterol-enriched SLBs (20 mol%) contained 1.4-fold more copies of spike^1268_Q3^ (mean λ_r_ = 10.0 ± 0.4) compared to cholesterol-free systems (mean λ_r_ = 7.0 ± 0.3) (Supplementary Fig. 15b, c). This cholesterol-mediated increase in oligomeric state directly correlates with our previous findings of elevated cluster density (1.5-fold increase in Fig. 4b) and enhanced membrane fusion efficiency (1.6-fold increase in Fig. 1f).

To determine whether cholesterol-dependent oligomerization of spike^1268^ persists in native cellular membranes. We expressed spike^1268_Q3^ in HEK293T cells and modulated membrane cholesterol levels by pre-treating the cells with or without MβCD and MβCD-CHO. Subsequently, SLBs were prepared from plasma membrane-derived vesicles of these cells, and subjected to the single-molecule photobleaching analysis (Fig. 5b, c). Consistently, the oligomeric state of spike^1268^ clusters exhibited a strong dependence on cellular cholesterol levels. Clusters derived from cholesterol-depleted membranes contained significantly fewer copies of spike^1268_Q3^ (λ_r_ = 8.0 ± 0.8) compared to those untreated membranes (λ_r_ = 10.0 ± 0.6) (Fig. 5d–g). Conversely, clusters from cholesterol-enriched membranes were comprised of significantly more copies of spike^1268_Q3^ (λ_r_ = 13.0 ± 1.3) (Fig. 5d–g). These coordinated results establish a mechanistic link between cholesterol-induced supramolecular assembly and biological function, suggesting that multivalent spike^1268^ oligomers serve as catalytically active fusion platforms.

### The C-terminus cytoplasmic domain of spike is critical for cholesterol-dependent function

Although coronavirus spike proteins are partially localized to detergent-resistant membrane (DRM) domains (in vitro correlates of dynamic lipid rafts),^32^ and ACE2 receptor enrichment in rafts is known to facilitate initial viral attachment,^33,34^ cholesterol dependence in SARS-CoV-2 entry may operate through a lipid raft-independent mechanism.^17^ The precise role of cholesterol in spike-mediated membrane fusion, particularly its compartment-specific regulation, remains incompletely defined. Our experimental data further revealed a significant correlation between the presence of cholesterol and the clustering intensity of spike^1268^ (Fig. 4a–c), suggesting a direct regulatory role of cholesterol on the oligomeric conformation of spike protein on the viral membrane.

**Fig. 6:**
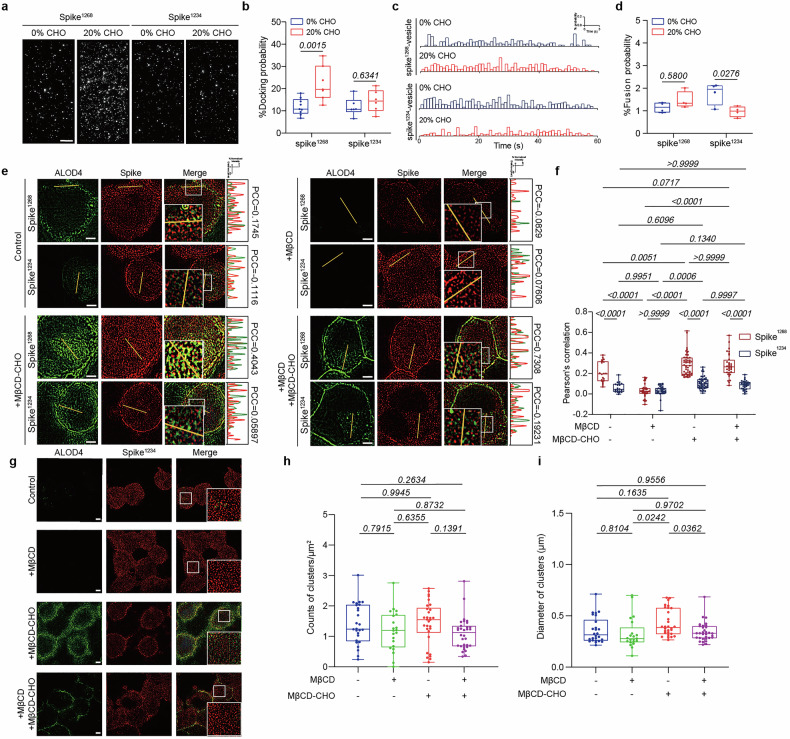
Truncated spike^1234^ exhibits impaired cholesterol responsiveness. **a** Representative fields of view of the fluorescent spots of ACE2-vesicles docking to corresponding spike-vesicles. The representative fields of view are docking events of ACE2-vesicles with corresponding spike-vesicles (0 or 20 mol% cholesterol). Scale bars, 10 μm. Scale bar applies to all images in this panel. **b** Box plots and data points show the docking probability corresponding to panel (**a**) from *N* ≥ 6 independent replicates. **c** Corresponding fusion histograms of ACE2-vesicles with spike^1268^-vesicles (upper) or spike^1234^-vesicles (lower). **d** Box plots and data points show the fusion probability to panel (**d**) from *N* ≥ 4 independent replicates. **e** The representative fields of spatial co-localization of spike^1268^/spike^1234^ protein (red) and cholesterol (green) on the plasma membrane (control, +MβCD, +MβCD-CHO, +MβCD + MβCD-CHO). Scale bars, 5 μm. Scale bar applies to all images in this panel. Corresponding fluorescence intensity profiles of spike^1268^/spike^1234^ protein (red) and cholesterol (green) along linear membrane regions in treated cell membrane are attached to the right of the fluorescence images. **f** Box plots and data points show comparative analysis of Pearson Correlation Coefficients (PCC) for colocalization between spike variants (spike^1268^ and spike^1234^, red) and cholesterol (green), across four cell membrane treatment groups corresponding to panel (**e**) from *N* ≥ 4 independent replicates. ROI = 20 (control), 35 (+MβCD), 50 (+MβCD-CHO), 32 (+MβCD + MβCD-CHO). **g** Reconstructed SIM images illustrating cholesterol-modulated spatial distribution of spike^1234^ clusters. Scale bars, 5 μm. Scale bar applies to all images in this panel. **h** Box plots and data points show the counts of clusters per μm^2^ for the spike^1234^ protein corresponding to panel (**g**) from *N* ≥ 4 independent replicates. **i** Box plots and data points show the size of clusters (Feret’s diameter) corresponding to panel (**g**) from *N* ≥ 4 independent replicates. Cluster quantification and diameter measurements were performed using NIS-Elements software, with ≥20 randomly selected cells in each group. CHO, cholesterol. In panels (**b**), (**d**), (**f**), (**h**) and (**i**), statistical analysis was performed using one-way (**h**, **i**), two-way (**d**) ANOVA followed by Tukey’s multiple comparisons test, or two-way ANOVA followed by Sidak’s multiple comparisons test (**b**, **f**)

To investigate the structural basis of cholesterol-mediated effects, we first employed cytoplasmic domain-truncated spike variants (spike^1234^; Supplementary Fig. 16a, b) in reconstituted fusion systems. Ensemble content mixing assays revealed that fusion kinetics for spike^1234^-vesicles followed a similar trend as spike^1268^ in the presence of Thr or 3C protease (Fig. 1c, d and Supplementary Fig. 16c, d). Moreover, in the single-vesicle fusion assay, cholesterol significantly potentiated spike^1268^-mediated vesicle docking (Fig. 6a, b) but exhibited no effect on spike^1234^-mediated vesicle docking. Conversely, single-vesicle fusion assays demonstrated mild inhibition of spike^1234^-mediated fusion activity (Fig. 6c, d). The superior docking and fusion efficiency of spike^1268^-vesicles over spike^1234^-vesicles at physiological cholesterol levels may result from a dual mechanism that cholesterol promotes vesicle docking for spike^1268^ via the interaction with the C-terminus cytoplasmic domain of spike, while it may suppress vesicle fusion by altering the membrane fluidity.^35,36^ Notably, the cholesterol on ACE2-vesicles had no significant effect on the bulk fusion efficiency at physiological levels of cholesterol (0–20 mol%), except for an inhibitory effect at 50 mol% cholesterol (Supplementary Fig. 17). Syncytium formation mediated by spike^1234^ showed no significant change under either cholesterol depletion or cholesterol repletion condition, indicating the C-terminal domain of the spike protein is critical for cholesterol-dependent membrane fusion (Supplementary Fig. 18).

We further quantified the correlation between cholesterol and spike proteins by conducting Pearson’s correlation coefficient (PCC) analysis, a standardized metric for evaluating spatial correspondence between fluorescence signals across independent imaging channels (Fig. 6e, f). As a control, transferrin receptor (TrfR) has no colocalization with cholesterol under any of the treatment conditions (Supplementary Fig. 19a, b). In untreated cells expressing spike^1268^ and spike^1234^ proteins, we observed moderate colocalization between spike^1268^ and cholesterol clusters, with the higher correlation coefficient of spike^1268^ (PCC = 0.21 ± 0.1) than spike^1234^ (PCC = 0.06 ± 0.05) (Fig. 6e, f). Subsequent cholesterol replenishment further enhanced the association of spike^1268^ with cholesterol-rich domains (PCC = 0.29 ± 0.10 upon repletion; PCC = 0.28 ± 0.11 upon rescue), while spike^1234^ consistently showed reduced colocalization across all conditions (PCC = 0.06–0.11) (Fig. 6e, f), suggesting that although spatial correlation analysis revealed cholesterol co-localization with spike^1268^, spike^1234^ maintained weak spatial correlation with cholesterol on both the untreated and cholesterol-sufficient cell membranes (Fig. 6e, f). Consistently, spike^WT1268^ also displayed stronger colocalization than spike^WT1234^ under untreated (0.22 ± 0.09 vs 0.06 ± 0.05), cholesterol-repletion (0.30 ± 0.09 vs 0.06 ± 0.06), and cholesterol-rescue conditions (0.28 ± 0.08 vs 0.07 ± 0.04) (Supplementary Fig. 20a, b). Note that, we also quantified the colocalization between spike protein and cholesterol using the Costes method with Manders’ coefficients (Supplementary Fig. 21a, b). Consistent with our previous conclusion, results from the Costes method confirmed strong colocalization between spike^1268^/spike^WT1268^ and cholesterol, while spike^1234^/spike^WT1234^ showed no such colocalization (Supplementary Fig. 21a, b).

In contrast to spike^1268^, the spatial distribution of spike^1234^ proteins remained unaffected by the cholesterol content of the cell membrane (Fig. 6g–i). Similarly, the number and diameter of spike^WT1234^ particles did not change regardless of plasma membrane cholesterol levels (Supplementary Fig. 22a–c), suggesting that the C-terminal cytoplasmic domain of spike protein serves as a cholesterol-sensing module for its localization and clustering on the viral membrane.

### Palmitoylation of CRR mediates cholesterol-dependent spike protein clustering and viral docking efficiency

**Fig. 7:**
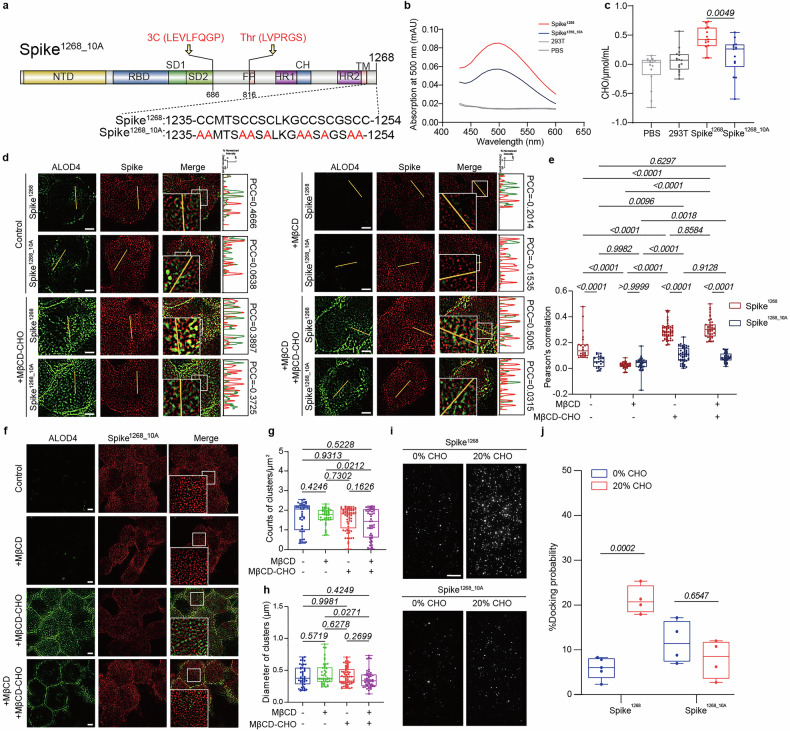
Cysteine-rich domain mediates cholesterol-dependent spike clustering. **a** Schematic representation of spike^1268_10A^, with ten cysteine-to-alanine substitutions (red). **b** Cholesterol binding assays: Representative absorbance spectra demonstrate direct interaction with spike^1268^ and spike^1268_10A^. **c** Quantification of cholesterol binding to spike^1268^ and spike^1268_10A^. **d** The representative fields of spatial co-localization of spike^1268^/spike^1268_10A^ protein (red) and cholesterol (green) on the plasma membrane (control, +MβCD, +MβCD-CHO, +MβCD + MβCD-CHO). Scale bars, 5 μm. Scale bar applies to all images in this panel. Corresponding fluorescence intensity profiles of spike^1268^/spike^1268_10A^ protein (red) and cholesterol (green) along linear membrane regions in treated cell membrane are attached to the right of the fluorescence images. **e** Box plots and data points show comparative analysis of PCC for colocalization between spike variants (spike^1268^ and spike^1268_10A^, red) and cholesterol (green), across four cell membrane treatment groups corresponding to panel (**d**) from *N* ≥ 4 independent replicates. ROI = 23 (control), 42 (+MβCD), 53 (+MβCD-CHO), 43 (+ MβCD + MβCD-CHO). **f** Reconstructed SIM images illustrating cholesterol-modulated spatial distribution of spike^1268_10A^ clusters. Scale bars, 5 μm. Scale bar applies to all images in this panel. **g** Box plots and data points show the counts of clusters per μm^2^ for the spike^1268_10A^ protein corresponding to panel (**f**) from *N* = 4 independent replicates. **h** Box plots and data points show the size of clusters (Feret’s diameter) corresponding to panel (**f**) from *N* ≥ 4 independent replicates. Cluster quantification and diameter measurements were performed using NIS-Elements software, with ≥20 randomly selected cells in each group. **i**. Representative fields of view of the fluorescent spots of ACE2-vesicles docking to corresponding spike-vesicles. The representative fields of view are docking events of ACE2-vesicles with corresponding spike-vesicles (0 or 20 mol% cholesterol). Scale bars, 10 μm. Scale bar applies to all images in this panel. **j** Box plots and data points show the docking probability corresponding to panel (**i**) from *N* = 4 independent replicates. In panel (**c**), statistical analysis was performed using t-test. In panels (**g**) and (**h**), statistical analysis was performed using one-way ANOVA followed by Tukey’s multiple comparisons test. In panels (**e**) and (**j**), Statistical analysis was performed using two-way ANOVA followed by Sidak’s multiple comparisons test

Palmitoylation at the cytoplasmic tail of viral spike proteins is a post-translational modification known to impact viral infectivity.^13^ Given the ten cysteine residues identified in the cytoplasmic tail of the SARS-CoV-2 spike protein, we suspected that palmitoylation of these CRR might regulate cholesterol-dependent spike clustering. To verify it, we generated a cysteine-deletion mutant (spike^1268_10A^) by replacing all ten cysteines with alanine to abolish palmitoylation in acyl-biotin exchange assays (Fig. 7a and Supplementary Fig. 23a). In addition, spike^1268_10A^ disrupted the binding to cholesterol in ALOD4-based cholesterol pull-down assays (Fig. 7b, c and Supplementary Fig. 23b).

To determine whether the loss of cholesterol binding in spike^1268_10A^ has functional deficits in cholesterol-dependent membrane fusion, we reconstituted the mutant of spike^1268_10A^ into ensemble content mixing assays (Supplementary Fig. 24a). In contrast to the cholesterol-dependent enhancement of fusion mediated by spike^1268^, varying cholesterol levels in spike-vesicle membrane had no significant effect on the fusion efficiency of spike^1268_10A^-vesicle and ACE2-vesicle (Supplementary Fig. 24b, c). Similarly, in cell–cell fusion assays, cholesterol modulation had no effect on syncytia formation mediated by the spike^1268_10A^ mutant (Supplementary Fig. 25a, b). As a control, cell viability and proliferation were not affected by different pre-treatments of cholesterol (Supplementary Fig. 26a, b). These results indicate that the CRR is essential for spike-mediated viral fusion.

Next, we examined the spatial distribution of spike^1268_10A^ on HEK293T cell membranes with modulated cholesterol levels using SIM. As a control, we did not observe any effect on the quantity of spike on the cell membrane by different cholesterol modulation (Supplementary Fig. 26c–f). To further investigate the cholesterol-binding specificity of spike^1268^, we compared its colocalization with spike^1268_10A^, under various cholesterol-modulated conditions (Fig. 7d, e). Quantitative analysis using PCC consistently showed higher colocalization of spike^1268^ with cholesterol across all treatments. In untreated membranes, the PCC values for spike^1268^ and spike^1268_10A^ were calculated to be 0.17 ± 0.10, and 0.05 ± 0.04, respectively (Fig. 7d, e). Cholesterol enrichment significantly enhanced the association of spike^1268^ with cholesterol (PCC = 0.29 ± 0.06 under cholesterol repletion and 0.31 ± 0.07 under cholesterol rescue), while the CRR mutant exhibited markedly reduced colocalization (PCC = 0.11 ± 0.06 and 0.09 ± 0.03, respectively) (Fig. 7d, e). And spike^1268_10A^ showed no changes in cluster density or size (Fig. 7f–h), in contrast to spike^1268^ (Fig. 4a–c), forming larger, denser clusters in cholesterol-containing membranes. In the vesicle-vesicle docking assays with reconstituted spike^1268^ or spike^1268_10A^, cholesterol-mediated enhancement of vesicle docking was abolished with spike^1268_10A^-vesicle (Fig. 7i, j). Together, these findings demonstrate that the cysteine-rich cytoplasmic domain and its palmitoylation are critical determinants of cholesterol-regulated SARS-CoV-2 infection.

To functionally validate the role of palmitoylation in viral infectivity, we treated SARS-CoV-2 pseudovirus with the palmitoylation inhibitor 2-bromopalmitate (2-BP) and evaluated its effect on infection efficiency in ACE2-expressing 293T cells. Pre-treatment with 2-BP resulted in a dose-dependent inhibition of pseudovirus infection (Supplementary Fig. 27), with an estimated IC_50_ of 12.98 μM (Supplementary Fig. 27a, b). This significant reduction in infectivity underscores the importance of palmitoylation for viral entry. Together, these findings demonstrate that CRR and its palmitoylation are critical determinants of cholesterol-regulated spike clustering and viral docking at host cell membranes, and that targeting this modification effectively disrupts SARS-CoV-2 infection.

## Discussion

To investigate the molecular mechanism of spike-mediated membrane fusion, we previously developed an in vitro ensemble cell-vesicle content-mixing assay to mimic SARS-CoV-2 invasion.^22^ In that assay, an engineered spike was expressed on the cell surface to mimic the viral membrane, while ACE2 was reconstituted on a group of liposomes to mimic the cell membrane. Although this system recapitulated key fusion intermediates, it exhibited limitations in specificity, manifesting as non-specific vesicle internalization. To address potential confounding effects from cell plasma membrane complexity and morphology difference from viral membrane, we refined the system to an in vitro single vesicle-vesicle fusion platform (Fig. 2). This optimized system significantly reduced non-specific endocytosis compared to the cell-vesicle system and minimized fusion machinery complexity while preserving spike’s fusogenic capacity (Fig. 1). Lipid composition, particularly cholesterol content, critically modulates membrane fusion efficiency by altering fluidity and membrane protein conformation.^27^ Cholesterol is essential for entry processes of multiple viruses, including HIV,^37^ Ebola,^38,39^ and influenza.^40^ Here, we extended the understanding of cholesterol’s function in spike-mediated membrane fusion by employing the vesicle-vesicle content-mixing assay (Fig. 1c–f). Our findings revealed that increasing cholesterol content in the membrane enhanced spike^1268^-mediated vesicle docking (Fig. 2a–d), whereas it had little effect on the fusion pore opening (Fig. 2f–h). This indicates that the cholesterol-mediated enhancement in bulk content mixing (Fig. 1e, f) primarily stems from promoted vesicle docking (Fig. 2b–d) rather than modulation of post-docking fusion steps (Fig. 2f–h). Similarly, in cell–cell fusion assays, cholesterol was required for high-efficiency syncytia formation by spike^1268^ and spike^WT^ (Fig. 3e, f and Supplementary Fig. 10a–c). Increasing cholesterol content in the spike-vesicle enhanced its fusion with ACE2 expressing cells (Supplementary Fig. 3a, b). Further pseudovirus infection assays revealed that cholesterol depletion markedly reduced infectivity, while cholesterol replenishment significantly enhanced it (Fig. 3g–i). We noticed that the apparent discrepancy between elevated membrane cholesterol in MβCD-CHO-treated cells (Fig. 3b and Supplementary Fig. 8) and the differential phenotypic outcomes across assays (Figs. 3, 4, 6, 7 and Supplementary Figs. 10, 14, 20, 21). We doubt this difference is due to the high baseline level of cholesterol (~40%) in the cell membrane.^26^ The baseline level of cholesterol is sufficient to support the formation of clusters of spike proteins, and thereby lead to maximal spike-mediated membrane fusogenicity. In this work, we utilized several reconstituted fusion assays to investigate the effect of cholesterol on spike-mediated viral fusion. A key insight from our multi-assay approach is the distinct time scales to reflect their underlying biological complexity of fusion process. Our reconstituted bulk and single-vesicle fusion assays are designed to capture the rapid, molecular-level kinetics of initial membrane merger between spike and ACE2-bearing membranes. In contrast, the cell–cell fusion and pseudovirus infection assay, which mimic the viral infection in physiological environment, requiring longer time of co-incubation, probes the slow, multi-step process of syncytia formation and viral infection. Our study provides compelling and multi-faceted evidence that cholesterol exerts its proviral effect specifically from the viral membrane compartment.

Previous studies indicated that reduced ACE2 binding of spike in cases of reduced cholesterol levels of the host cell membrane.^33,34,41,42^ Another elegant physiological study^41^ demonstrates that MβCD application potently inhibits viral infection. This co-exposure means the antiviral effect likely results from the combined depletion of cholesterol from both the host membrane and the viral membrane. Our data, which isolates the variable of host membrane cholesterol alone, specifically demonstrate that the loss of viral membrane cholesterol is the dominant factor restricting fusion. This conclusion is further supported by another study,^42^ which shows that HP-β-CD exerts far stronger antiviral effects when targeting virus particles (IC_50_: 0.35–0.53 mM) compared to host cells (IC_50_: 4.99–5.2 mM), providing strong complementary evidence supporting that viral membrane cholesterol is the dominant regulator of SARS-CoV-2 entry. Moreover, our results show that analogous modulation of cholesterol in the ACE2-presenting membranes has minimal impact on spike-mediated fusion across all assays, even a mild inhibition at high cholesterol concentrations (50 mol%) (Supplementary Figs. 2, 7, 11 and 12). Thus, our results address complementary aspects of viral entry regulation by cholesterol, suggesting that the cholesterol-dependent enhancement of viral fusion is exclusively driven by increased cholesterol in the spike-bearing membrane compartment, rather than the host membrane.

Cholesterol may modulate membrane protein conformation via membrane curvature, phase behavior, or directly interact with transmembrane proteins via motifs like cholesterol-recognition amino acid consensus (CRAC-CARC) motifs.^43^ However, the CRAC-CARC within the transmembrane domain of SARS-CoV-2 spike protein does not exhibit direct interaction with cholesterol.^18^ Building upon previous findings that cholesterol modulates the oligomerization and membrane organizing activity of the SARS-CoV fusion peptide,^19^ providing a structural basis for calcium-mediated promotion of fusion peptide insertion,^20^ and enhancing fusion peptide-mediated membrane merger,^21^ our study demonstrated that cholesterol acts through the CRR to regulate multiple steps of fusion. Specially, N-SIM imaging revealed cholesterol-dependent clustering of spike^1268^ (Fig. 4a–c and Supplementary Fig. 14a–c). Quantitative single-molecule photobleaching analysis revealed that cholesterol-enriched membranes supported spike clusters with significantly higher oligomeric copies compared to cholesterol-free or cholesterol-depleted membranes (Fig. 5d–g and Supplementary Fig. 15b, c), demonstrating cholesterol-driven supramolecular assembly. Truncating the cytoplasmic domain (spike^1234^) abolished the enhancement effect in cell–cell fusion (Supplementary Fig. 18a, b), and attenuated the association with cholesterol (Fig. 6e, f and Supplementary Figs. 20a, b and 21a, b) and cholesterol-dependent clustering (Fig. 6g–i and Supplementary Fig. 22a–c), pinpointing this region as a cholesterol-sensing module. Sequence analysis identified a conserved CRR within the cytoplasmic domain. CRR palmitoylation is critical for viral packaging, fusion, and infectivity,^12,13^ and palmitoylated proteins typically localize to cholesterol-rich lipid rafts.^44^ In our studies, alanine-substituted CRR mutated variant spike^1268_10A^ lost cholesterol association and cholesterol-dependent functions in viral fusion (Fig. 7 and Supplementary Figs. 24b, c and 25a, b). These results implicated CRR as the interaction hub for cholesterol to play a stimulatory role in viral infection.

Given the persistent challenges in containing viral transmission and mitigating long-term public health burdens, developing broad-spectrum inhibitors targeting conserved regions is imperative. Our results proposed two complementary approaches: cholesterol-lowering interventions and the inhibition of CRR palmitoylation. Early observational data from COVID-19 patients showing lower 28-day mortality with statins suggested cholesterol-lowering statins might benefit COVID-19 outcomes.^45^ Although the later high-quality studies, including a large randomized controlled trial, have failed to confirm a definitive therapeutic benefit.^46^ Notably, our in vitro use of MβCD to manipulate cholesterol is not translatable to in vivo applications, as MβCD exhibits strong hemolytic activity. However, structurally related cyclodextrins with improved safety profiles, including 2-hydroxypropyl-β-cyclodextrin (HP-β-CD) and sulfobutylether-β-cyclodextrin (SBE-β-CD), which act by depleting cholesterol to disrupt viral entry, have been approved for human use.^33,41,47–49^ The inhibition of CRR palmitoylation blocks cholesterol-dependent spike clustering (Fig. 7g–i) and pseudovirus infection (Supplementary Fig. 27a–c), leveraging its high conservation across variants and minimal interference with receptor-binding domains. Previous studies highlighted S-CRD peptides targeting the CRR of spike as pan-coronavirus inhibitors.^14^ Our work further suggests targeting CRR palmitoylation, which remains highly conserved across SARS-CoV-2 variants, could block cholesterol-dependent spike clustering. This approach achieves dual suppression by simultaneously disrupting spatial organization of viral fusion machinery and mechanical modulation of membrane fusion dynamics.

**Fig. 8:**
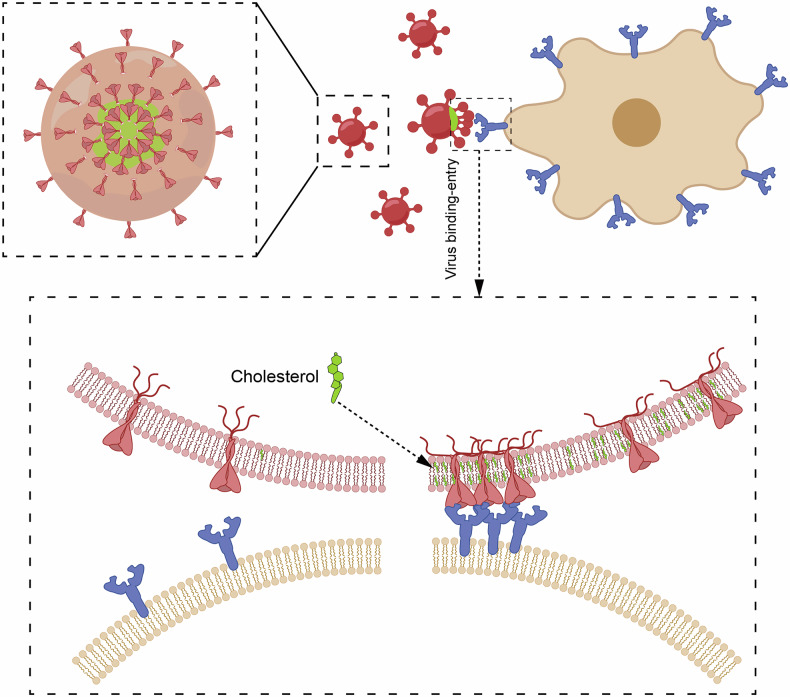
Mechanistic model of cholesterol-induced spike clustering facilitates viral membrane fusion. Cholesterol enrichment facilitates the lateral reorganization of spike glycoproteins into oligomeric cluster conformations through specific interactions with the cysteine-rich domain in the C-terminal cytoplasmic tail. This structural remodeling may drive the formation of fusion-active nanodomains on host cell membrane. The spatial nanoscale reorganization could promote spike-mediated viral-host docking efficiency by multivalent ACE2 engagements, and lower the activation energy barrier for viral membrane fusion with host cell. Importantly, cholesterol-mediated spatial confinement may optimize the geometrical alignment of spike trimers, enabling synchronized conformational transitions from prefusion to postfusion states. The figure was created using Adobe Illustrator

In summary, our studies elucidate that cholesterol on the viral membrane may serve as membrane fusion platform for SARS-CoV-2 spike-mediated viral entry. At such active fusion sites, spike protein forms into an oligomeric cluster via a specific interaction between palmitoylated CRR in C-terminal cytoplasmic domain and cholesterol. This spatial reorganization increases spike-host docking efficiency and viral uptake (Fig. 8). These insights advance our understanding of cholesterol’s regulatory role in viral entry and highlight CRR palmitoylation as a promising target for coronavirus therapeutics.

## Materials and methods

### Plasmid construction

The genes encoding wild-type full-length spike^WT^ (20-1273, based on the SARS-CoV-2 reference sequence, GenBank accession number MN908947), spike^WT1268^ (20-1268), spike^WT1234^ (20-1234), spike^1268^ (20-1268), spike^1234^ (20-1234) were cloned into the PTT5 vector as described in previous work.^22^ For the mutants spike^1268^ and spike^1234^, the residues within the S1/S2 cleavage site (PRRAR) were replaced with the 3C cleavage site (LEVLFQGP), and the residues within the S2’ cleavage site (KPSKR) were replaced with the Thr cleavage site (LVPRGS). The extracellular soluble domain sequence of human ACE2 (a.a.19-615) with N-terminal GP67 signal peptide was cloned into pFastBac1 by using previously described methods.^22^

### Cell culture and transient transfection

HEK293T, Expi293F, and Sf9 cells were obtained from the American Type Culture Collection (ATCC) and cultured as described in previous work.^22^

For transient transfection, HEK293T cells were seeded in 6-well culture plates at a density achieving 70–90% confluency within 24 h. One hour prior to transfection, the cell supernatant was replaced with 3 mL supplementary medium (Dulbecco's modified Eagle’s medium (DMEM) containing 2% fetal bovine serum (FBS)) in each well 1 h before transfection. Transfection complexes were prepared by mixing 3 μg/well plasmid encoding the target protein and 9.75 μg/well polyethylenimine (PEI; 1 mg/mL) transfection reagent in a 1:3.25 (w/w) ratio in 300 µL of antibiotic-free DMEM, followed by a 15-min incubation at room temperature. Then the mixture was gently added dropwise to each well. Cells were incubated at 37 °C in the CO₂ incubator for 24 h. Cell supernatant was replaced with fresh DMEM and continued to cultivate for an additional 24 h. Following three rounds of phosphate-buffered saline (PBS) washes, cells were collected for downstream applications.

### Protein production and purification

We used the same constructs and protocols to purify spike^1268^, spike^1234^ proteins, and ACE2 protein as previously described.^22^ The protein sample concentrations were measured by ultraviolet light absorption at 280 nm, and aliquots were flash-frozen in liquid nitrogen and stored at −80 °C.

ALOD4 protein was expressed, purified, and fluorescently labeled based on a previously described protocol.^31^ Briefly, the plasmid pALOD4 (Addgene # 111026) was transformed into BL21 (DE3) pLysS competent cells (Tsingke). After induction of expression by 1 mM isopropyl β-D-1-thiogalactopyranoside (IPTG) at 18 °C for 16 h, cells were harvested by centrifugation at 3800 × *g* for 15 min. Cell pellets were resuspended with lysis buffer (50 mM Tris-HCl, pH 7.5, 1 mM Tris (2-carboxyethyl) phosphine (TCEP), 1 mM phenylmethylsulfonyl fluoride (PMSF)) and lysed by a high-pressure homogenizer (AH-NANO, ATS engineering limited). ALOD4-His×6 was purified using NTA-Ni resin (Smart-lifesciences) for Ni chromatography. A protein solution with ~15 mM NaCl concentration was loaded on a pre-equilibrated 5-mL Source 15Q anion exchange column (Cytiva) for ion exchange chromatography operations. Subsequently, soluble proteins were purified via size-exclusion chromatography using Superdex 200 Increase 10/300 gel filtration column (Cytiva). The purified protein was further concentrated by 10 kDa Amicon Ultra centrifugal filters (Millipore). Purified ALOD4 proteins were confirmed by 15% SDS-PAGE. For long-term storage, the concentrated protein with 20% (v/v) glycerol was flash-frozen in liquid nitrogen and stored at −80 °C.

For ALOD4 labeled with cyanine 3-maleimide (Cy3-maleimide) (Goyoobio), ALOD4 was mixed with Cy3-maleimide at 4 °C for 16 h. The reaction was quenched by adding 10 mM dithiothreitol (DTT). Excess dye was removed by desalting. Following the conjugation reaction, unconjugated dye removal was achieved via size-exclusion chromatography using a PD-10 Desalting Column (Cytiva) packed with Sephadex G-25 resin. Prior to sample loading, the column was pre-equilibrated with PBS. The ALOD4-Cy3 mixture was eluted by gravitational flow, with the labeled protein fraction collected in the initial 3.5 mL PBS elute, while unbound dye was retained in the column. Final protein concentration and labeling efficiency were determined by spectrophotometric analysis with the molar extinction coefficients ε280 = 26,420 M⁻¹ cm⁻¹ for ALOD4 and ε550 = 150,000 M⁻¹ cm⁻¹ for Cy3.

### Vesicle reconstitution

For the ensemble content mixing assay, the lipid composition of the spike-vesicle was 45 mol% PC, 20 mol% PE, 15 mol% PS, and 20 mol% CHO. For ACE2-vesicle, the lipid composition was supplemented with 9% DGS-NTA (1,2-dioleoyl-sn-glycero-3-[(N-(5-amino-1-carboxypentyl) iminodiacetic acid) succinyl] (nickel salt)) by replacing the corresponding molar percentage of PC based on the lipid composition of the spike-vesicle.

For the single vesicle-vesicle lipids mixing assay, the lipid composition of the spike-vesicle was 42.5 mol% PC, 20 mol% PE, 15 mol% PS and 20 mol% CHO, 1 mol% 1-oleoyl-2-(12-biotinyl(aminododecanoyl))-sn-glycero-3-phosphoethanolamine (Biotin-PE), and 1.5 mol% 1,1′-dioctadecyl-3,3,3′,3′-tetramethylindodicarbocyanine perchlorate (DiD). The lipid composition of the ACE2-vesicle was 42 mol% PC, 20 mol% PE, 15 mol% PS and 20 mol% CHO, 2 mol% 1,1′-dioctadecyl-3,3,3′,3′-tetramethylindocarbocyanine perchlorate (DiI). For the single vesicle-vesicle content mixing assay, the lipid component of the spike-vesicle and ACE2-vesicle were similar to that of the ensemble content mixing, except that 1.5 mol% DiD/DiI was replaced by a corresponding molar percentage of POPC. All the lipids were sourced from Avanti Polar Lipids.

For the vesicle reconstitution, dried lipid films that were prepared by evaporating chloroform from a lipid mixture under a nitrogen stream and then incubated overnight under a vacuum, were dissolved in vesicle buffer (20 mM HEPES, 100 mM NaCl) to a final lipid concentration of 10 mM. After ten freeze-thaw cycles, unilamellar vesicles were extruded through polycarbonate filters (50 nm pore size, Avanti Polar Lipids) at least 31 times. For the preparation of spike-vesicle, purified 1 μM spike proteins were mixed with 50 μL unilamellar vesicles, which were supplemented with 0.8% OG and the concentration of OG was kept at 0.8% during the reconstitution. After 4 °C incubation for 20 min, the incubated protein-lipid mixture was diluted two-fold with detergent-free vesicle buffer so that OG concentration in the system was lower than the critical micelle concentration (~0.6%). The mixtures were then dialyzed against vesicle buffer to remove the detergent at 4 °C overnight. Detergent-free spike-vesicle was confirmed by 10% SDS-PAGE.

To test the orientation of reconstituted spike protein, the spike^1234^-vesicle and spike^1268^-vesicle were digested with 2 w/v % pepsin (Sigma) at 37 °C for 30 min. Equal amounts of the vesicles with digestion or not were subjected to 10% SDS-PAGE and then transferred to 0.22 μm PVDF membrane for western blot analysis. Spike protein was detected by anti-S2 primary antibody (1A9) (GeneTex) described as previously described.^22^

The ACE2-vesicle was reconstituted as previously described in detail.^22^ The size distributions of the spike-vesicle and ACE2-vesicle were analyzed by negative-staining transmission electron microscopy imaging as described previously.^22^

### Ensemble vesicle-vesicle content mixing assays

For the ensemble vesicle-vesicle content mixing assays, 20 mM self-quenched SRB (Sigma) as a content indicator was encapsulated into ACE2-vesicle. Content mixing was measured in the vesicle buffer based on an increase in fluorescence emission at 585 nm upon excitation with a 532 nm laser light. The dequenching signal resulted from dilution of the initially self-quenched SRB upon fusion between ACE2-vesicle and spike-vesicle. All content-mixing experiments were performed in the presence of 10 units/mL (U) 3C proteases (Beyotime) or 5 U Thr (Sigma). The fusion efficiency was calculated by the real-time fluorescence intensity increase of SRB in the content mixing assay divided by the maximum intensity of SRB obtained by adding 0.1% Triton X-100 (Sigma). The fluorescence emission of the vesicle-vesicle fusion assay was recorded with a Cary Eclipse Fluorescence Spectrophotometer (Agilent) at ambient temperature (~25 °C).

### Single vesicle-vesicle docking assays

PEG-coated flow chambers were prepared following a previously described protocol.^50^ Briefly, a quartz slide and glass coverslip were passivated by coating the surface with methoxy polyethylene glycol (mPEG, Laysan Bio) molecules that contain 0.1 wt% biotin-PEG (Laysan Bio) to alleviate non-specific binding of vesicles.^29^ A quartz slide was assembled into a flow chamber and incubated with 0.1 mg/mL neutravidin for 30 min.

The single vesicle-vesicle lipid mixing assay was performed with DiD-labeled spike-vesicle and DiI-labeled ACE2-vesicle. The biotin-PE containing spike-vesicle was immobilized on a PEG-coated quartz surface via the specific interaction between biotin and neutravidin. After 30 min incubation, free spike-vesicle was removed by three rounds of wash with vesicle buffer. The DiI-containing ACE2-vesicle was injected into the flow chamber at ambient temperature (~25 °C) for 15 min. After three rounds of wash with vesicle buffer, 5-10 images were taken stochastically upon simultaneous excitation with 532 nm and 640 nm laser light. The docking efficiency was calculated by the number of colocalized ACE2-vesicle divided by the total number of spike-vesicles immobilized on the chamber surface. When vesicles dock but do not fuse, DiI (donor) and DiD (acceptor) remain confined to their respective membranes, resulting in minimal energy transfer and low FRET efficiency. In contrast, upon lipid mixing, the dyes diffuse into the other vesicle via a single continuous membrane, enabling energy transfer from DiI to DiD and causing a measurable increase in FRET efficiency.The lipid mixing is quantified using FRET efficiency (E),

$${\rm{E}}=\frac{{I}_{A}}{{I}_{D}+{I}_{A}}$$

Where *I*_*A*_ is the acceptor (DiD) intensity, *I*_*D*_ is the donor (DiI) intensity. We define a “high-FRET population” as events with E > 0.5, indicating significant lipid mixing (over 50% energy transfer). We have included these details into the method section in the revised manuscript.

### Single vesicle-vesicle fusion assays

The single vesicle-vesicle content mixing assays were performed with unlabeled spike-vesicle and ACE2-vesicle encapsulated SRB. Following the same protocol as the single vesicle-vesicle docking assays, after ACE2-vesicle docking with spike-vesicle, 0.5 U/μL Thr protease was added to the chamber to initiate the spike-mediated fusion process. Fusion events between spike-vesicle and ACE2-vesicle were monitored for 1 min upon 532 nm laser light excitation. The fusion events were identified, and the fusion probability was calculated as previously described.^29^

In general, fusion events were specifically identified by stepwise increases in SRB fluorescence intensity, a signal that arises from the dequenching of SRB dye upon mixing of the internal contents of docked ACE2-vesicles and spike-vesicles. Fusion probability was quantified using the following formula:

$$Fusion\,probability=\frac{Nfused}{Ndocked}\times 100{\boldsymbol{ \% }}$$

*N*docked: Total colocalized ACE2-vesicles. *N*fused: ACE2-vesicles showing stepwise increases after SRB dequenching across the time-lapse imaging period.

### Syncytia formation assay

The syncytia formation assay was performed as described in previous work.^22^ Briefly, spike-EGFP-cell (4 × 10^4^ cells/well) were co-cultured with ACE2-cell (2 × 10^4^ cells/well) in a 2:1 ratio. The cells were seeded on the poly-L-lysine coated 96-well plate (BIOFIL) in a total volume of 100 μL DMEM supplemented with 5% lipid-depleted fetal bovine serum (VivaCell), 100 U/mL penicillin, and 100 μg/mL streptomycin. After the cells had fully adhered, the co-cultures were scanned in real-time every 2–3 h for up to 48 h using the IncuCyte S3 system at 20× magnification. The size of the syncytia (GFP-positive area) was measured using Image J. A total of 60 syncytia were randomly selected and measured in each condition, with the data derived from at least 3 independent biological replicates.

In the syncytia formation assays, cholesterol depletion was carried out by pre-treating spike-EGFP cells with 1 mM MβCD (Sigma) for 1.5 h at 37 °C, while cholesterol repletion was achieved by treating the cells with 1 mM MβCD-conjugated cholesterol (Sigma) for 1.5 h at 37 °C. Following these treatments, the pretreated spike-EGFP cells were co-cultured with ACE2 cells to assess syncytia formation. For the detection of the cholesterol on cell membranes, 3 μM ALOD4 was added to cells in each well and incubated for 30 min at 37 °C.

For western blot analysis, cell lysates were obtained by homogenizing the cells in RIPA buffer (50 mM Tris, 150 mM NaCl, 1% Triton X-100, 1% sodium deoxycholate, 0.1% SDS, pH 7.4; Sigma) supplemented with 1 mM PMSF (Sangon Biotech) using a probe sonicator (300 W power setting, 3 cycles of 5 s sonication-10s rest on ice). The lysates were then centrifuged at 15,000 rpm for 20 min at 4 °C. Total protein concentrations were determined by using the BCA protein assay kit (Yeasen). Equal amounts of protein (25 μg) were loaded onto 15% SDS-PAGE and transferred to 0.22 μm polyvinylidene difluoride (PVDF) membrane (Millipore). To minimize non-specific binding, the membrane was blocked with 5% w/v skim milk diluted in TBST (20 mM Tris, 137 mM NaCl, 0.1% Triton X-100, pH 7.6; BOSTER) for 1 h at room temperature. ALOD4 was detected with 0.8 μg/mL anti-6×His antibody (Huabio). In addition, the membrane was incubated overnight at 4 °C with 400 ng/mL mouse monoclonal anti-β-actin antibody (ZSGB-BIO). After three rounds of wash with TBST, the membrane was incubated with 80 ng/mL HRP-conjugated goat anti-rabbit/mouse IgG(H + L) (ZSGB-BIO) diluted in 5% w/v skim milk in TBST for 2 h at room temperature. Following another three washes with TBST, the immunoblot images were captured by chemiluminescent HRP substrate (Millipore) using a Tanon-5200 Chemiluminescent Imaging System (Tanon Science & Technology).

### Pseudovirus infection assay

293T cells were co-transfected with pNL4–3.luc.RE (the luciferase reporter-expressing HIV-1 backbone) and PTT5-SARS-CoV-2-spike (encoding for spike protein) using lipo8000 (Beyotime Biotechnology, China). Pseudovirus particles were efficiently released in the supernatant. The supernatant was harvested at 72 h post-transfection, centrifuged at 3000 × *g* for 10 min, and frozen to −80 °C. To detect the effect of cholesterol or 2-BP on the infection of pseudovirus, ACE2 expressing cells were plated at a density of 10^4^ cells per well in a 96-well plate one day prior to infection. Pseudovirus was mixed with an equal volume of 2-BP, MβCD, or MβCD-CHO solutions, which was serially diluted in DMEM at room temperature for 30 min. The mixture was transferred to the ACE2 expressing cells. After 12 h, the medium was replaced, and incubation was continued for an additional 48 h. Viral infection efficiency was quantified by measuring luciferase activity using the Luciferase Assay System (Promega) according to the manufacturers instructions.

### Single-molecule photobleaching assay

For single-molecule photobleaching assay, we employed both synthetic vesicles and native membrane-derived nanoparticles. The lipid composition of the spike-vesicle was 65 mol% or 45 mol% PC, 15 mol% PE, 15 mol% PS, 0 mol% or 20 mol% CHO, 5 mol% 1,2-dioleoyl-sn-glycero-3-phosphoethanolamine-N-[methoxy(polyethylene glycol)-2000] (PEG2000-PE, Avanti Polar Lipids), and 0.001 mol% DiI.

For native membrane nanoparticles, HEK293T cells expressing Q3-tagged spike protein were treated with 1 mM MβCD, or 1 mM MβCD-CHO for 1 h at 37 °C to modulate cholesterol levels. The cells were then labeled with Cy5-cadaverine (0.4 mM) using gpTGase in accordance with previously established protocolscitation,^51^ with specific adaptations: labeling was performed in DMEM containing 12 mM CaCl₂, 1 mg/mL BSA, and 50 ng/µL gpTGase for 25 min at 4 °C. After washing with cold DPBS, the labeled cells were subjected to ultrasonic disruption (100 W) to produce membrane nanoparticles.

Quartz slides were carefully cleaned by sequential sonication in 5% Alconox, acetone, and 1 M KOH, extensively rinsed with deionized water, Piranha cleaning (a 7:3 mixture of sulfuric acid and hydrogen peroxide), and finally, extensively rinsed with deionized water. The supported lipid bilayer (SLB) was formed by incubating the small unilamellar vesicles or native membrane nanoparticles with the slides. Cy5-labeled spike proteins were excited by red (632 nm) laser light. The monomers of the labeled spike proteins that bound to DiI-labeled SLB were counted at an excitation power of 3 mW. The quantification of spike monomers within individual fluorescent spots was accomplished by sequential stepwise photobleaching events occurring within a single fluorescent spot. Histograms represented the distribution of the number of photobleaching steps, which corresponded to the spike monomers per fluorescent spot. The distribution of the observed numbers of labeled monomers per fluorescent spot was fitted to a Poisson distribution, assuming a random binding process. And the “real” Poisson distribution function that includes both labeled and unlabeled spike monomers was calculated as previously described.^52^

### Preparation of SIM samples

Prior to SIM sample preparation, HEK293T cells expressing spike protein underwent identical cholesterol depletion/repletion pretreatment as the syncytia formation assay using 1 mM MβCD and 1 mM MβCD-conjugated cholesterol. For cholesterol rescue, MβCD-treated cells were subsequently incubated with 1 mM MβCD-conjugated cholesterol.

For dual-color imaging of cholesterol and spike, pre-treated HEK293T cells expressing spike protein at ~70% confluence were initially incubated with 3 μM Cy3-labeled ALOD4 in PBS containing 1% BSA at 37 °C for 1 h, followed by three rounds of wash with PBS. Cholesterol detection was achieved through Cy3-labeled ALOD4 staining. Cells underwent fixation with 4% paraformaldehyde (PFA, Servicebio) at room temperature for 1 h to ensure complete immobilization and prevent spike protein redistribution, with subsequent three rounds of wash with PBS. Fixed cells were then incubated with 4 μg/mL spike-specific monoclonal primary antibody at 4 °C overnight. After two PBS washes, samples were incubated with 1.33 μg/mL APC-labeled goat anti-mouse IgG secondary antibody (minimal cross-reactivity; 1:150 dilution in PBS) for 2 h at room temperature, followed by three PBS washes. 10 μL anti-fade reagent (Beyotime) was applied to a 25 × 75 mm microscope slide (Citotest). The cell-seeded coverslip (φ 14 mm) was carefully mounted onto the slide for permanent sealing.

### 3D-SIM super-resolution imaging

N-SIM imaging was performed on a Nikon Ti-2 microscope (Nikon, Japan) equipped with a 100×/1.45 NA objective lens. Samples were imaged in three-dimensional structured illumination microscopy (3D-SIM) mode. Dual-laser excitation was implemented with 561 nm (15 mW/30 mW adjustable) and 647 nm (30 mW) lasers for cholesterol (Cy3) and spike protein (APC) detection, respectively. The optical path incorporated a dichroic mirror beam splitter and an EMCCD camera (Photometrics, Cascade II) for signal acquisition.

Each fluorescent channel required 15-frame acquisition at 800 ms exposure per cell. Merged dual-channel images were typically captured within 1 min. This configuration minimized x-y/z-axis drift through rapid acquisition and robust sample immobilization.

Image processing involved two stages: Initial reconstruction of raw SIM data was performed using NIS-Elements software (Nikon) with reconstruction algorithm (Reconstruct Slice), applying three core parameters: Illumination Modulation Contrast (IMC), High-Resolution Noise Suppression (HRNS), and Section Out-of-Focus Blur (SOFB). Standard parameters included IMC = 0.63 and HRNS = 1.25 for both channels, with channel-specific SOFB = 0.26/0.4 for Cholesterol (561 nm), and SOFB = 0.45 for Spike protein (647 nm). All fluorescence images were analyzed with NIS-Elements software (Nikon).

### Quantitative image analysis for microscopy-based methods

Image quantification was performed using established protocols. For syncytium formation assays, the area of cell–cell fusion was quantified using ImageJ software. Images were first calibrated based on the scale bar derived from original micrographs. Regions corresponding to syncytia (identified by expanded EGFP fluorescence resulting from membrane fusion) were manually outlined, and the area was calculated for each structure.

For cluster analysis, fluorescent clusters were quantified using the Automated Measurement function in NIS-Elements software (Nikon). A consistent threshold was applied across all images within an experiment to distinguish clusters from background signals, generating a binary layer for analysis. The software automatically quantified cluster count, area, and Feret diameter (Supplementary Fig. 28). All detected clusters and corresponding quantitative data were exported for further statistical analysis.

### Statistical analysis

In general, NIS-Elements, ImageJ, Prism and Adobe Illustrator (Figs. 1a, b, 2a, e, 3a, b, 5a, b, 7a and 8) software were used to analyze the data. Statistical significance was determined by either Student’s t-test or ANOVA (one-way/two-way) as specified in corresponding figure legends. The threshold for statistical significance was set at *p* < 0.05, with *p* ≥ 0.05 indicated as non-significant (n.s.).

For the analysis of Costes method with Manders’ coefficients, five colocalization states were defined to distinguish the positional relationships. Isolation (0 < M1/M2 ≤ 0.1, minimal to no signal overlap), Weak connection (0.1 < M1/M2 ≤ 0.23, minimal edge contact without obvious overlap), Partial overlap (0.23 < M1/M2 ≤ 0.3, moderate signal overlap), Middle overlap (0.3 < M1/M2 ≤ 0.4, significant signal overlap), and High overlap (0.4 < M1/M2 ≤ 0.8, extensive signal coincidence). M1 represents the proportion of spike protein localized in cholesterol-enriched regions, and M2 denotes the proportion of cholesterol regions covered by spike protein.

## Supplementary information

Supplemental material

## Data Availability

All data that support the findings of this study are available at 10.6084/m9.figshare.30903542. Materials are available from the corresponding author. Source data are provided with this paper.
