# Supplementary material for: Molecular mechanism of cholesterol-dependent membrane fusion in SARS-CoV-2 entry
Source: Signal Transduct Target Ther. 2026 Feb 17;11:57. doi: 10.1038/s41392-026-02573-z (PMC12909914; doi:10.1038/s41392-026-02573-z)
Supplement: Supplementary file 1 — Supplemental material [file 41392_2026_2573_MOESM1_ESM.docx]

Supplementary Materials for

Molecular mechanism of

cholesterol-dependent membrane fusion in SARS-CoV-2 entry

Wei Li^1 #^, Mengdan Wu^1#^, Shirong Feng^1#^, Jiaming Su^2#^, Qian Niu^3#^, Weijun Lin^1^, Jiaqi Fan^1^, Lele Cui^1^, Yijuan Xiang^1^, Hao Li^1^, Kaiyu Li ^1^, Zhaoming Su^1^, Guangwen Lu^4^, Ying Lai^1^*

Correspondence to: [ylai@scu.edu.cn](mailto:ylai@scu.edu.cn)

^#^ These authors contributed equally to this work

**This PDF file include**

Materials and Methods

Figures. S1 to S28

Materials and Methods

### Cryo-EM sample preparation.

### Spike-vesicles and ACE2-vesicles in this experiment were reconstituted according to the previously described method of vesicle reconstitution. Then, a total of 3 μL of the vesicle solution was applied onto glow-discharged (50 s) 200-mesh R2/1 Quantifoil Cu grids. The grids were blotted for 2.5 s in 100% humidity with no blotting offset and rapidly frozen in liquid ethane using a Vitrobot MarkIV (Thermo Fisher).

### Cryo-EM sample data acquisition and data processing.

The frozen grids of vesicle samples were loaded into a Titan krios G4 (Thermo Fisher) operated at 300 kV, with a Falcon 4i direct electron detector and a Selectris energy filter operated in zero-loss mode with a slit width of 10 eV. Microscope magnification was at 165,000× (corresponding to a calibrated sampling of 0.75 Å per physical pixel) with a total accumulated dose of 40 e^−^/Å^2^ per micrograph, using EPU software (Thermo Fisher). Micrographs were lowpass filtered accordingly and displayed in EMAN2.31.

# Ensemble cell-vesicle content mixing assays

For the ensemble cell-vesicle content mixing assays, 20 mM self-quenched SRB (Sigma) as a content indicator was encapsulated into spike-vesicle. The dequenching signal resulted from dilution of the initially self‐quenched SRB upon fusion between the spike-vesicles and ACE2 expressing cells (1-2×10^6^) in the presence of 5 U Thr. Content mixing was measured in the vesicle buffer based on an increase in fluorescence emission at 585 nm upon excitation with a 532 nm laser light, recorded with a Cary Eclipse Fluorescence Spectrophotometer (Agilent) at ambient temperature (~25 °C).

**Cytotoxicity assay**

For the cytotoxicity of 2-BP, MβCD, and MβCD-CHO toward spike-expressing and ACE2-expressing cells, we used the Cell Counting Kit-8 (CCK-8; Beyotime Biotechnology). Briefly, cells were seeded into 96-well plates at a density of 1×10^4^ cells per well and incubated at 37 °C for 12-15 hours to allow adherence. The culture medium was then replaced with DMEM containing 2-BP, MβCD, or MβCD-CHO at corresponding concentrations. Spike-expressing cells were cultured at 37°C for 1.5 hours, while ACE2 expressing cells were cultured at 37 °C for 48 hours. After this period, 20 μL of CCK-8 solution was added to each well, followed by a 4-hour incubation. Cell viability was determined by measuring absorbance at 450 nm using a microplate reader (Moleculardevices CMax Plus).

**Membrane fraction preparation**

Cells transiently transfected with the plasmid expressing spike were pretreated with MβCD and MβCD-CHO, and harvested after 48 hours. After washing with ice-cold PBS and centrifugation, the cell pellet was resuspended in PBS supplemented with 0.5 mM TCEP and 1 mM PMSF. Cells were lysed using a high-pressure homogenizer, and the resulting lysate was centrifuged at 10,000 rpm at 4°C for 10 minutes. The supernatant was subsequently subjected to ultra centrifugation at 35,000 rpm at 4°C for 1 hour. The pellet, containing the crude membrane fraction was collected and resuspended to form a homogeneous solution, followed by Western blot analysis to detect spike protein.

**Flow cytometry measurement**

Flow cytometry measurement was performed as previously described. Briefly, 1×10^6^ HEK293T cells that were transfected with plasmid encoding specified protein were collected, washed and incubated for 30 min at 4 °C with 5 µg/mL anti S2 primary antibody (1A9) (GeneTex). After washing with PBS, cells were incubated with 500 ng/mL APC‐labeled goat anti‐mouse IgG secondary antibody (Biolegend) for 30 min at 4 °C. To determine the expression level of ACE2, 1×10^6^ cells were incubated with 1.07 µg mL−1 rabbit polyclonal anti‐ACE2 primary antibody (SinoBiological), followed by incubating with 1.88 µg/mL Alexa Fluor 488 labeled goat anti rabbit IgG (H+L) secondary antibody. All samples were washed with 500 µL PBS for three times to remove the nonspecific binding antibodies and were monitored by FCM. The NovoExpress software (ACEA Biosciences) was used to analyze the data.

Figure. S1.


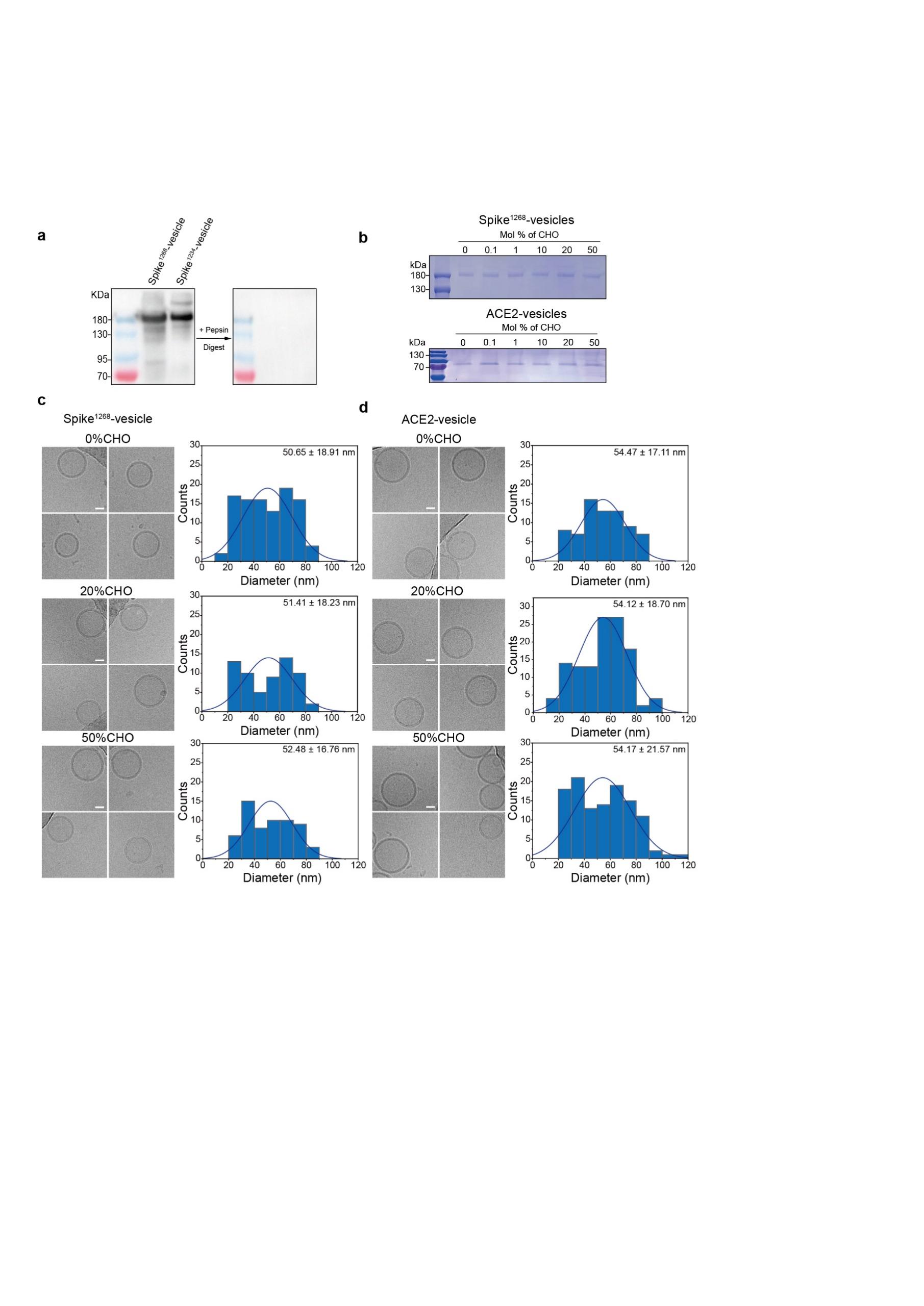


**Fig. S1. Reconstitution of ACE2-vesicle and** **spike^1268^-vesicle. a.** Surface orientation analysis of spike^1268^ and spike^1234^ protein on vesicles via pepsin digestion followed by anti-RBD immunoblotting. **b.** SDS-PAGE of spike-vesicles (0-50 mol% cholesterol) and ACE2-vesicle (0-50 mol% cholesterol). **c.** Cryo-EM images of spike^1268^-vesicle containing 0 mol% cholesterol, 20 mol% cholesterol and 50 mol% cholesterol, with corresponding diameter distribution histogram. **d.** Cryo-EM images of ACE2-vesicle containing 0 mol% cholesterol, 20 mol% cholesterol and 50 mol% cholesterol, with corresponding diameter distribution histogram. Scale bars, 20 nm. Scale bar applies to all images in this panel. CHO, cholesterol.

Figure. S2.


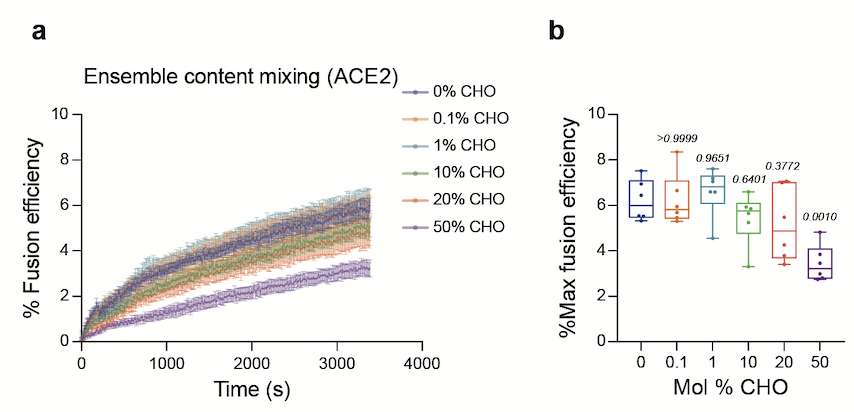


**Fig. S2. Fusion efficiency is insensitive to cholesterol levels of ACE2-vesicles in the ensemble vesicle-vesicle content mixing system.** **a.** The ensemble vesicle-vesicle content mixing of cholesterol titration experiment with ACE2-vesicles (0-50 mol% cholesterol) fused with spike^1268^-vesicles (20 mol% cholesterol). Traces represent the mean ± SEM from N=6 independent replicates. **b.** Box plots and data points show the maximum fusion efficiency corresponding to panel a. CHO, cholesterol. Statistical analysis was performed using one-way ANOVA followed by Tukey’s multiple comparisons test.

Figure. S3.


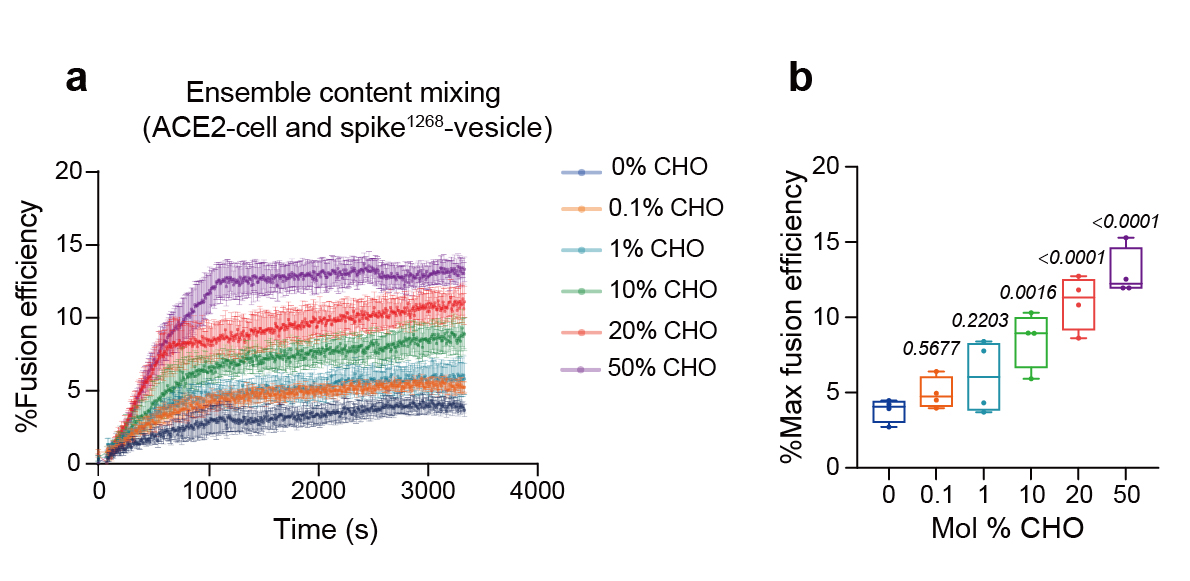


**Fig. S3. Cholesterol-dependent membrane fusion mediated by spike protein in a reconstituted cell-vesicle content-mixing system.** **a.** The ensemble content mixing of cholesterol titration experiment with spike^1268^-vesicles (0-50 mol% cholesterol) fused with ACE2 cell. Traces represent the mean ± SEM from N=4 independent replicates. **b.** Box plots and data points show the maximum fusion efficiency corresponding to panel **a.** CHO, cholesterol. Statistical analysis was performed using one-way ANOVA followed by Tukey’s multiple comparisons test.

Figure. S4.

**
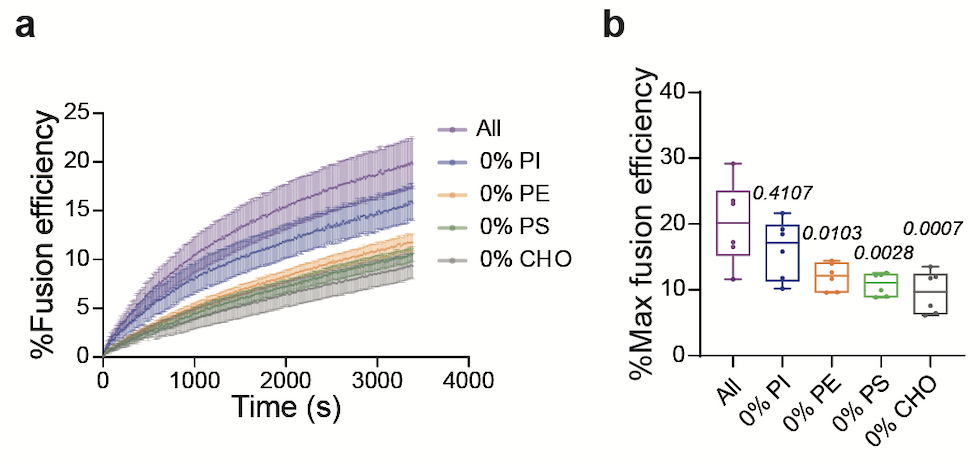
**

**Fig. S4. The effect of different lipids on spike^1268^-mediated ensemble content mixing. a.** Ensemble content mixing of vesicles with different lipid composition. Membrane composition was systematically modified by depletion of phosphatidylinositol (PI), phosphatidylethanolamine (PE), phosphatidylserine (PS), or cholesterol (CHO). The lipid composition of the All vesicles: 37.5 mol% PC, 7.5 mol% PI, 20 mol% PE, 15 mol% PS, and 20 mol% cholesterol. Traces represent the mean ± SEM from N=6 independent replicates. **b.** Box plots and data points show the maximum fusion efficiency corresponding to panel **a.** CHO, cholesterol. Statistical analysis was performed using one-way ANOVA followed by Tukey’s multiple comparisons test.

Figure. S5.


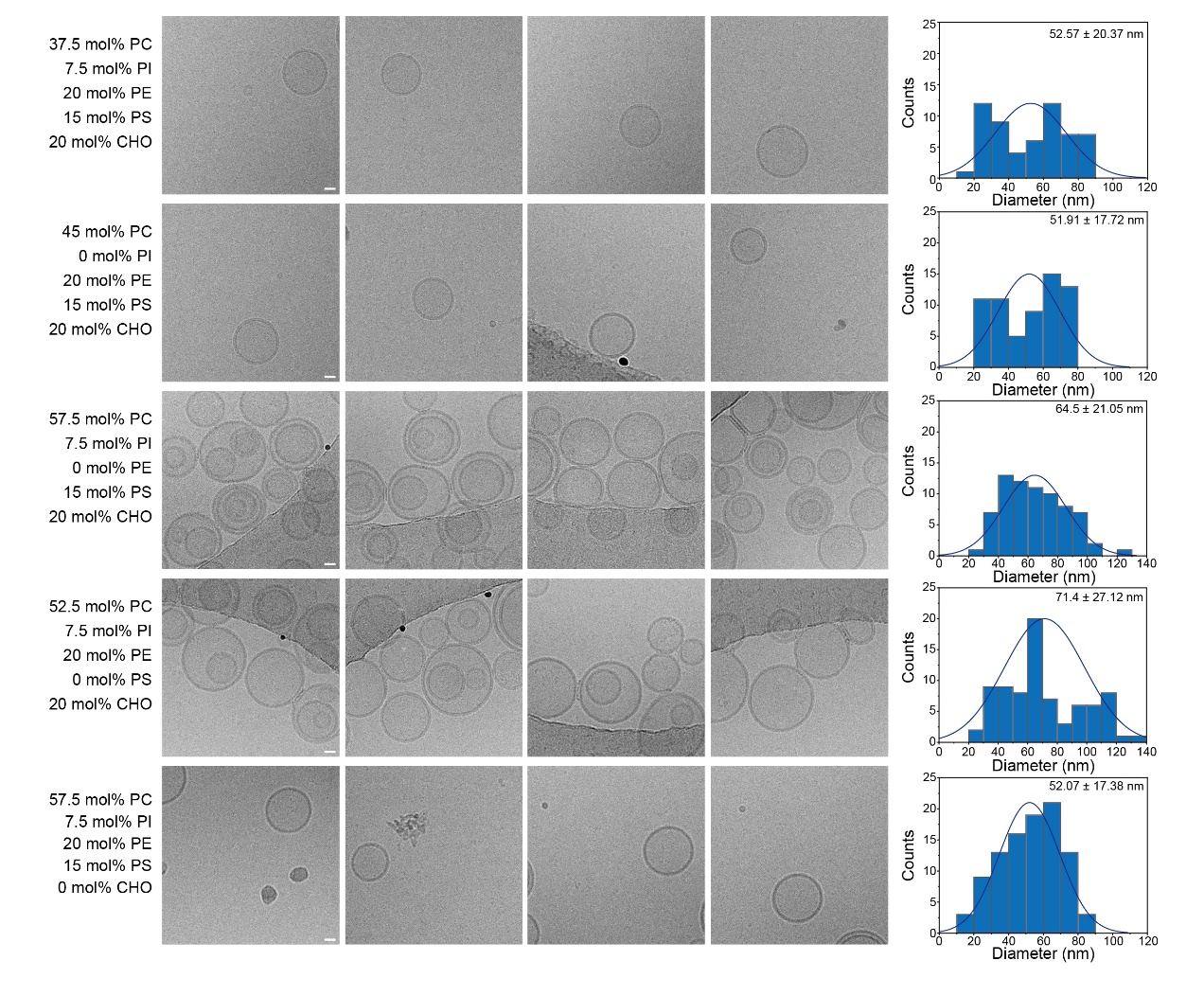


**Fig. S5. Cryo-EM images of vesicle with different lipid composition.** Complete lipid composition of vesicle (37.5 mol% PC, 7.5 mol% PI, 20 mol% PE, 15 mol% PS, 20 mol% cholesterol). For PI-deficient, PE-deficient, PS-deficient, and cholesterol-deficient vesicle, corresponding lipid was replaced by the same molar percentage of POPC. Scale bar: 20 nm. Scale bar applies to all images in this panel. CHO, cholesterol.

Figure. S6.

**
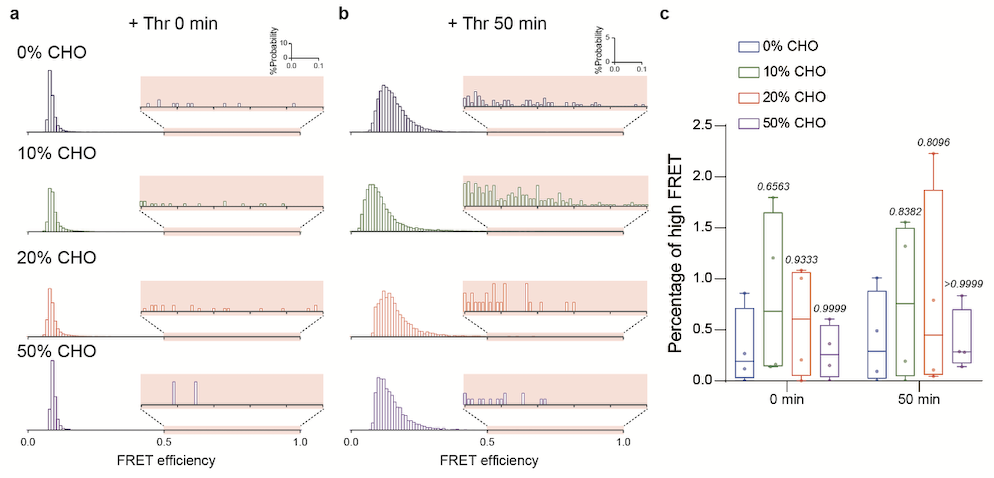
**

**Fig. S6. Cholesterol-dependent vesicle fusion dynamics in single vesicle lipid-mixing assay. a.** The FRET distribution diagram between spike^1268^-vesicle (0-50 mol% cholesterol) and ACE2-vesicle (20 mol% cholesterol) at 0 min post-Thr protease activation. **b.** The FRET distribution diagram at 50 min post-Thr protease activation. **c.** Quantification of high-FRET populations from panel **b.** CHO, cholesterol. Statistical analysis was performed using two-way ANOVA followed by Tukey’s multiple comparisons test.

Figure. S7.


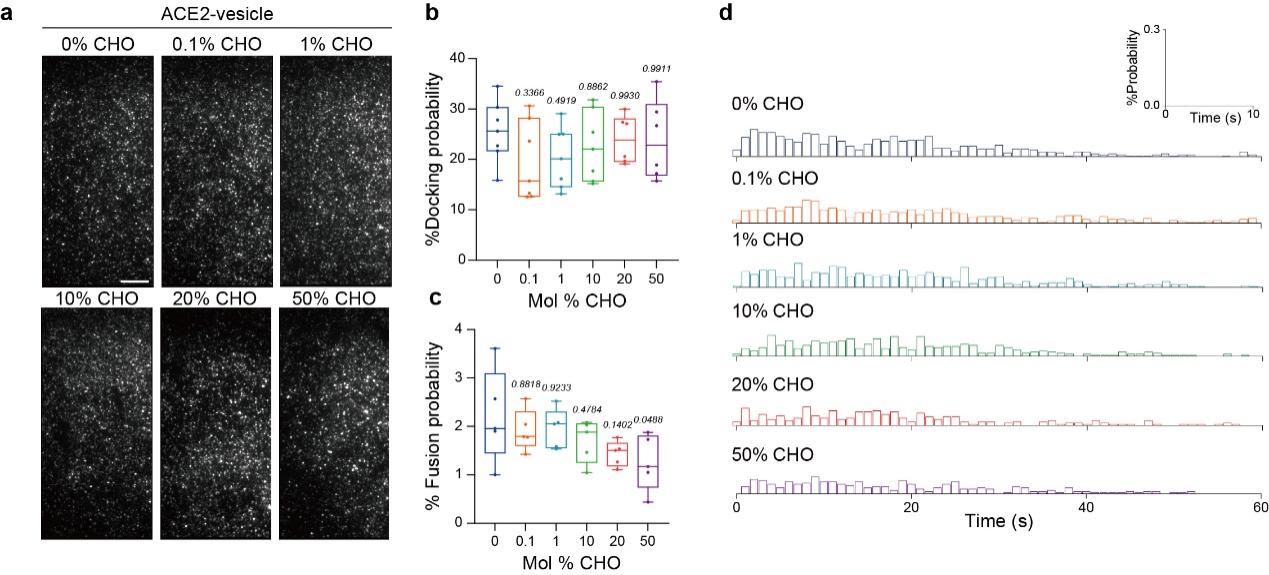


**Fig. S7. The cholesterol on ACE2-vesicle does not affect the single-vesicle docking and fusion process.** **a.** Representative fields of view of the fluorescent spots of ACE2-vesicle docking to the spike-vesicle. The representative fields of view are docking events of ACE2-vesicle (0, 0.1, 1, 10, 20 and 50 mol% cholesterol) with spike^1268^-vesicle (20 mol% cholesterol). Scale bars, 10 μm. Scale bar applies to all images in this panel. **b.** Box plots and data points show the docking probability corresponding to panel **a** from N≥5 independent replicates. **c.** Box plots and data points show the fusion probability of ACE2-vesicle (0, 0.1, 1, 10, 20 and 50 mol% cholesterol) with spike^1268^-vesicle (20 mol% cholesterol) from N=5 independent replicates. **d.** Fusion histograms corresponding to panel **c**. CHO, cholesterol. In panels **b** and **c**, statistical analysis was performed using one-way ANOVA followed by Tukey’s multiple comparisons test.

Figure. S8.


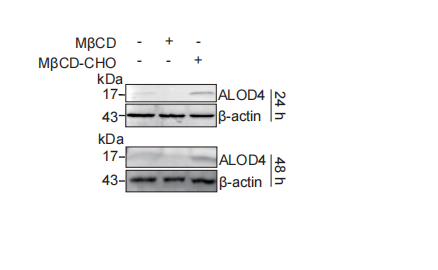


**Fig. S8. Quantification of cholesterol level on cell membranes under different treatments at specified time points.** Western blot analysis of cholesterol in the plasma membrane by ALOD4 after 24h or 48h of treatment. Cell membrane was treated with MβCD or MβCD-CHO to reduce or replenish cholesterol content on the plasma membrane.

Figure. S9.


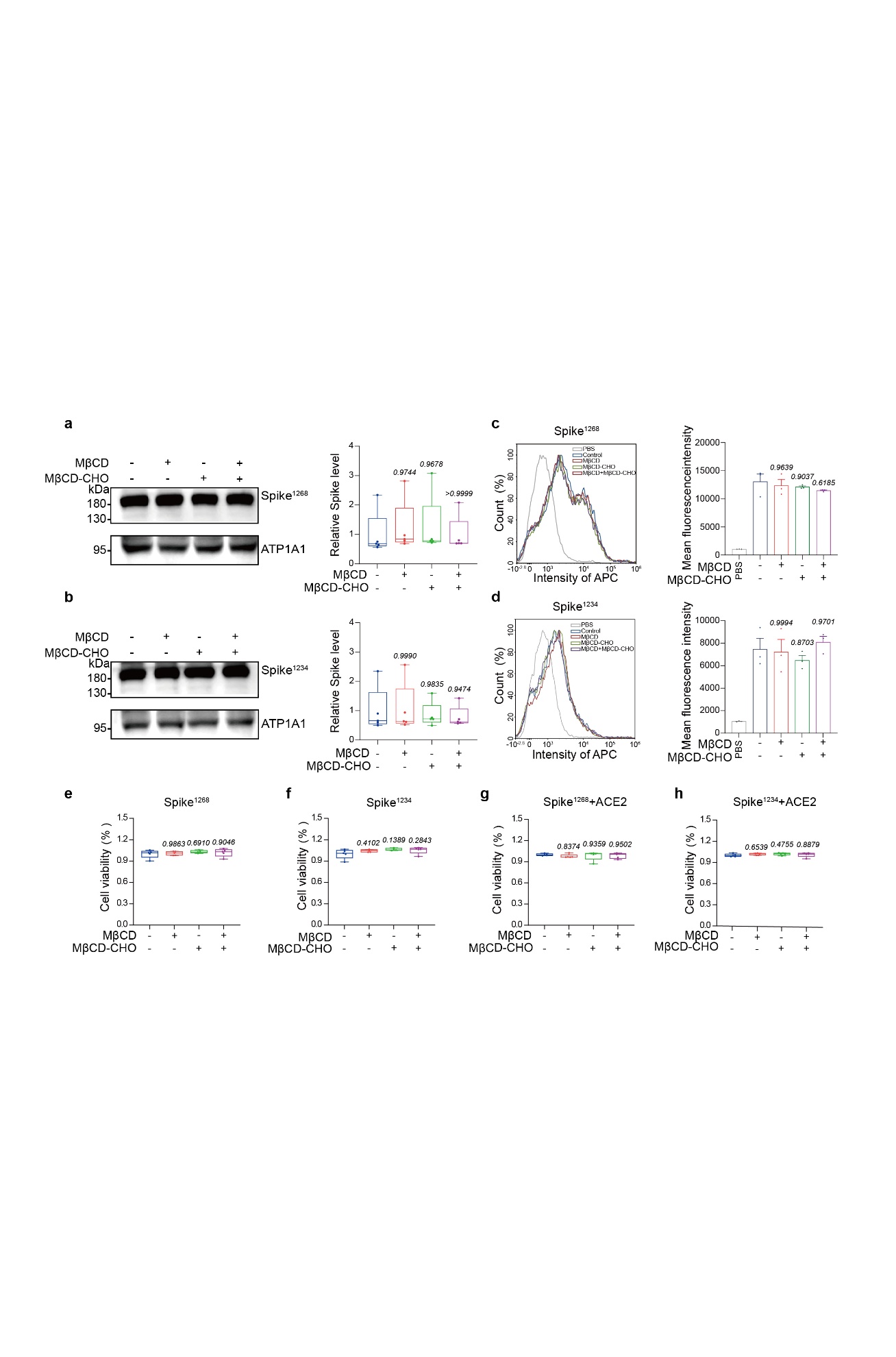


**Fig. S9. Quantification of membrane spike protein levels and assessment of cell viability with different treatments. a-b.** Immunoblots of spike protein on membrane fraction demonstrating consistent partitioning of spike^1268^ **a** and spike^1234^ **b**, with quantification analysis of spike^1268^, spike^1234^, following corresponding treatments, from N=5 independent replicates. **c-d,** Flow cytometry analysis of spike protein expression on cell membrane of spike^1268^ **c** and spike^1234^ **d** following corresponding treatments, with mean fluorescence intensity values summarized from N=3 independent replicates. CCK-8 assays assessing cell viability following corresponding treatments for spike^1268^-GFP-cells **e**, spike^1234^-GFP-cells **f**. **g**. CCK-8 measurements after 48-hour coculture with ACE2 under the same treatment conditions for spike^1268^-GFP-cells **g** and spike^1234^-GFP-cells **h**. In panels **a-h**, statistical analysis was performed using one-way ANOVA followed by Tukey’s multiple comparisons test.

Figure. S10.


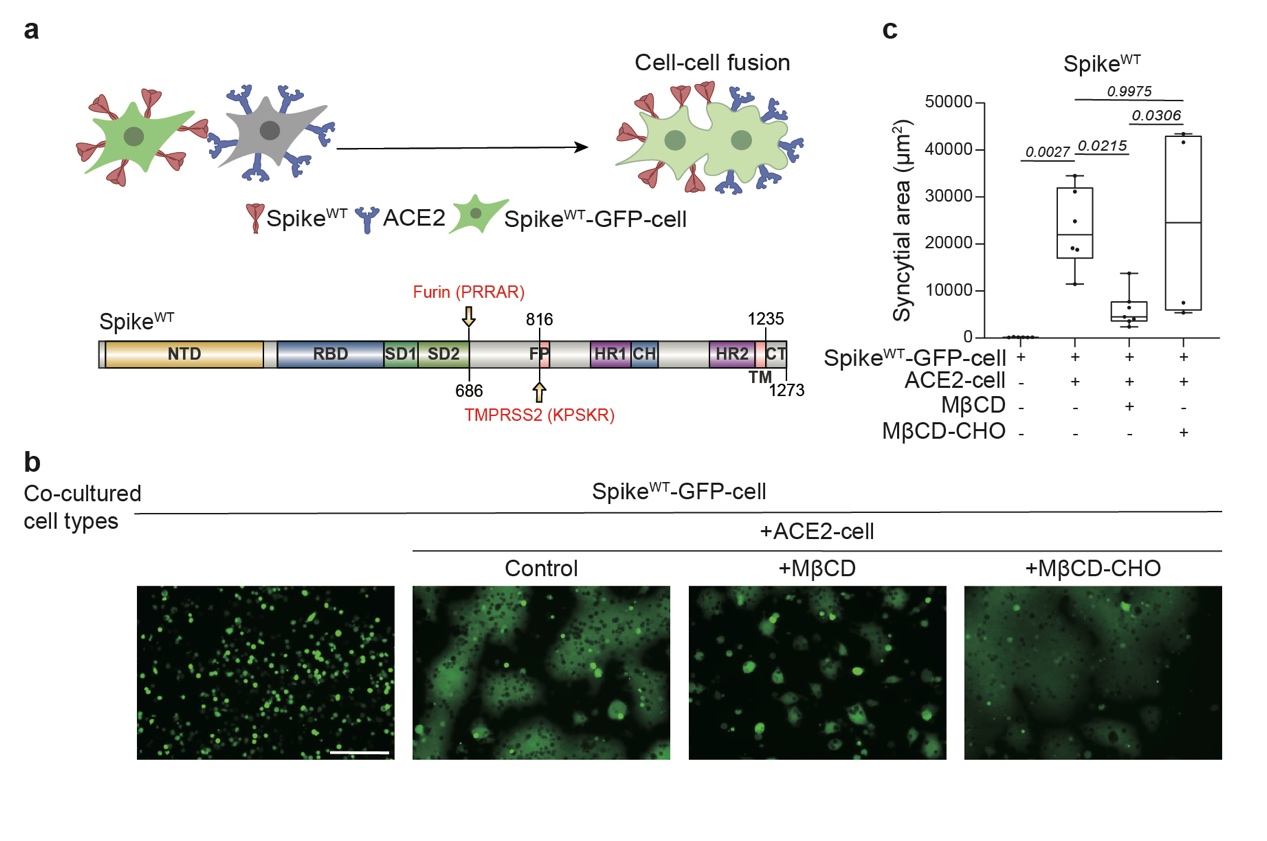


**Fig. S10. Cholesterol enhances spike^WT^-mediated syncytium formation. a.** Schematic diagram of the cell-cell fusion assay using ACE2-cell and spike^WT^-EGFP-cell. Domain architecture of spike^WT^ with functional motifs highlighted. **b.** Syncytia formation was induced by cell-cell fusion under cholesterol-depleted or replenished spike^WT^-EGFP-cell. Scale bar, 200 μm. Scale bar applies to all images in this panel. **c.** Box plots and data points show the syncytial area in cell-cell fusion corresponding to panel **b.** from N≥4 independent replicates. Statistical analysis was performed using one-way ANOVA followed by Tukey’s multiple comparisons test.

Figure. S11.


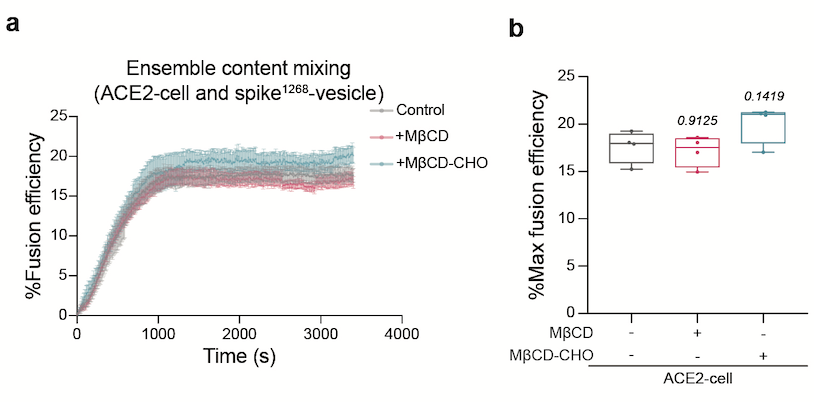


**Fig. S11. Fusion efficiency is insensitive to cholesterol levels of ACE2-vesicles in ensemble cell-vesicle content mixing system. a.** The ensemble cell-vesicle content mixing assay of the spike^1268^-vesicle with ACE2-cell. ACE2 cells were pretreated with MβCD, MβCD-CHO, and non-treated (control). Traces represent the mean ± SEM from N=4 independent replicates. **b.** Box plots and data points show the maximum fusion efficiency corresponding to panel **a**. Statistical analysis was performed using one-way ANOVA followed by Tukey’s multiple comparisons test.

Figure. S12.

**
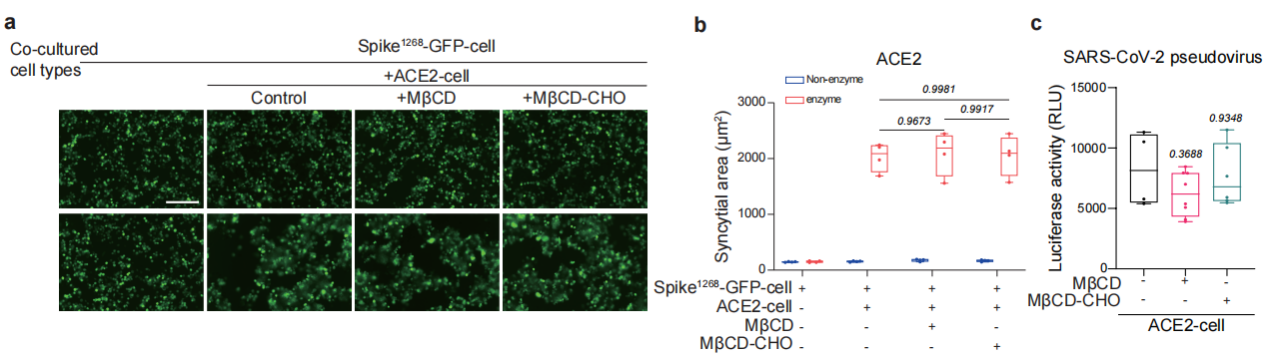
**

**Fig. S12. Adjustment of Cholesterol on ACE2-cell membrane has no impact on cell-cell fusion or pseudovirus entry.** **a.** Syncytia formation induced by ACE2 and spike^1268^-mediated cell-cell fusion. ACE2 cells were pretreated with MβCD or MβCD-CHO. Scale bar, 200 μm. Scale bar applies to all images in this panel. **b.** Box plots and data points show the syncytial area in cell-cell fusion corresponding to panel **a** from N=4 independent replicates. **c.** SARS‐CoV-2 pseudovirus infection in hACE2-expressing 293T cells that were either left untreated (control) or pre-treated with MβCD or MβCD-CHO. Luciferase activity was measured to quantify the infection. In panel **b**, statistical analysis was performed using two-way ANOVA followed by Tukey’s multiple comparisons test. In panel **c,** statistical analysis was performed using one-way ANOVA followed by Tukey’s multiple comparisons test.

Figure. S13.


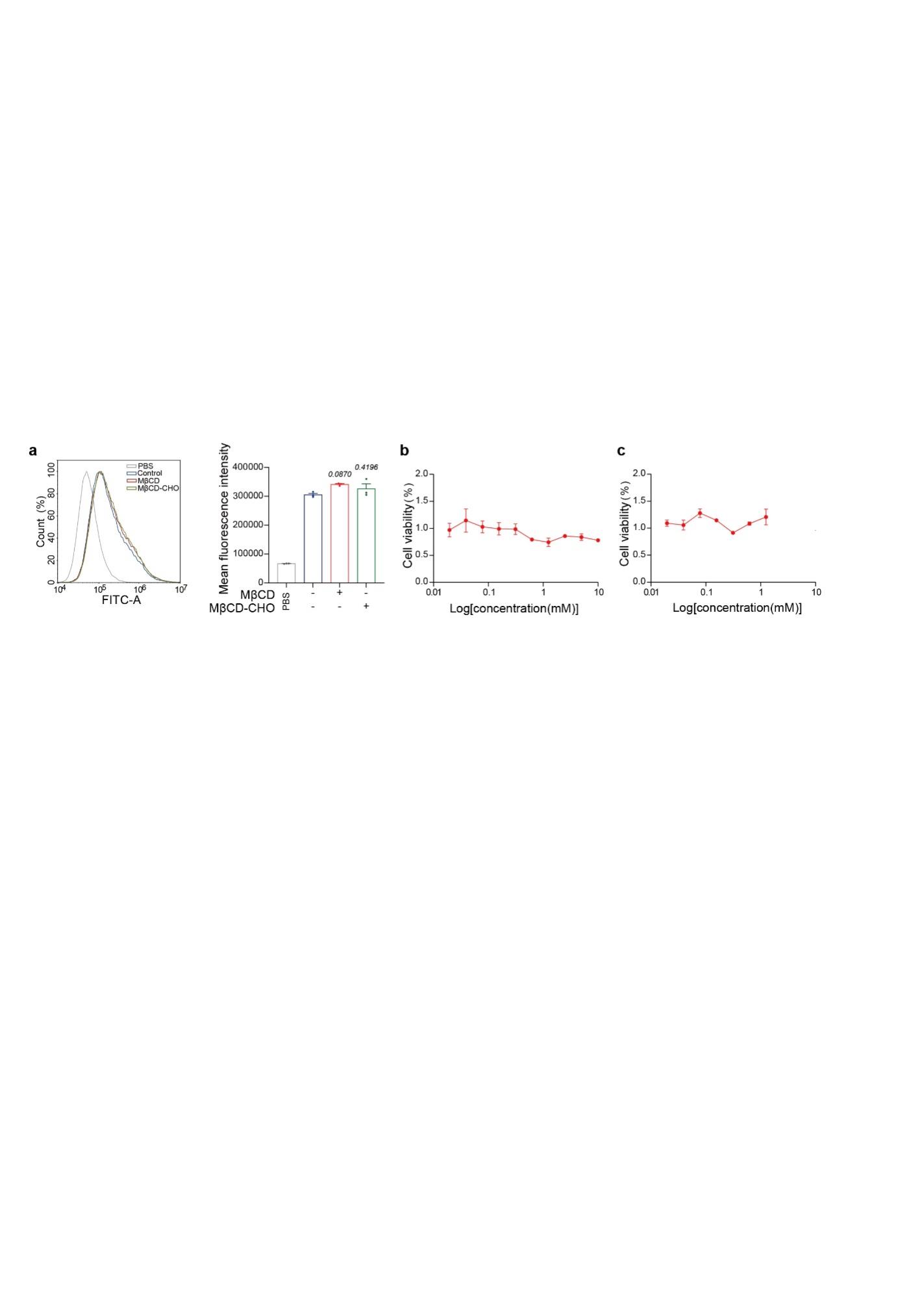


**Fig. S13. Quantification of membrane ACE2 protein levels and assessment of cell viability with different treatments of cholesterol. a.** Flow cytometry analysis of ACE2 protein expression on cell membrane of ACE2-expressing cells (293T-ACE2) following corresponding treatments, with mean fluorescence intensity values summarized from N=3 independent replicates. **b, c.** CCK-8 assays assessing cell viability for ACE2 expressing 293T cells with 0-10 mM MβCD (**b**) or 0-1.25 mM MβCD-CHO (**c**) pretreatment.

Figure. S14.


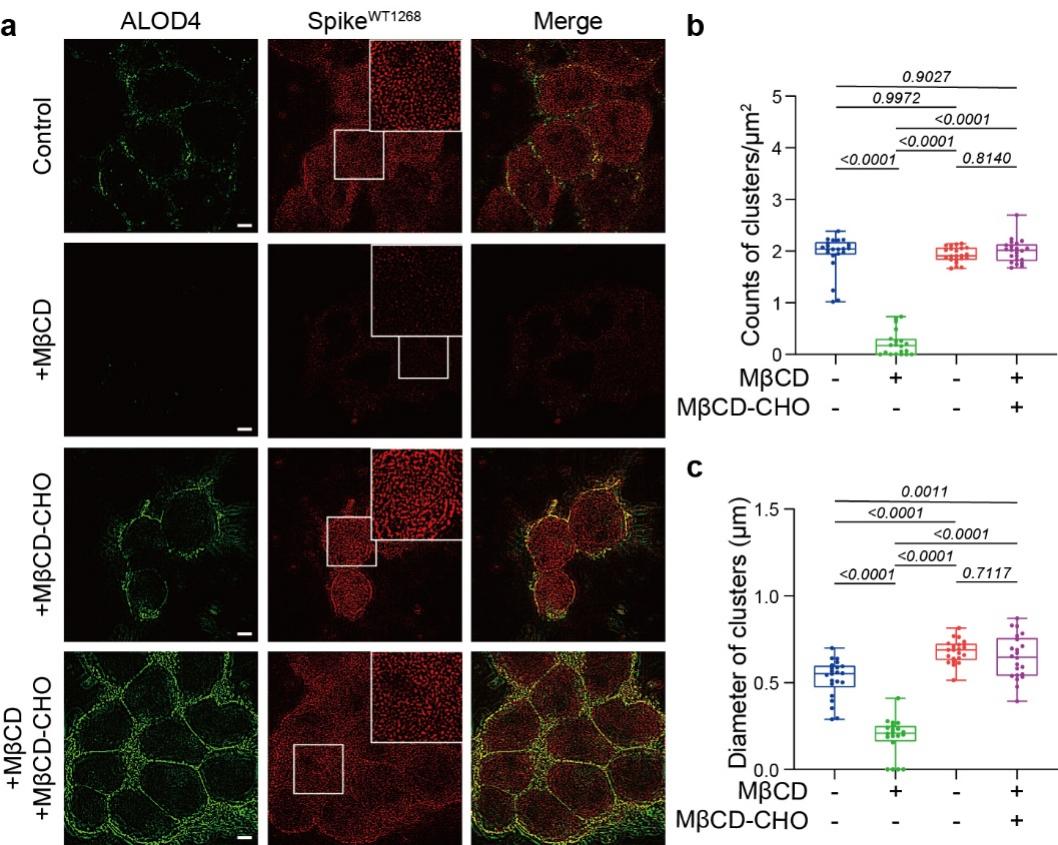


**Fig. S14. Cholesterol orchestrates nanoscale clustering of spike^WT1268^ protein. a.** Reconstructed SIM images illustrating cholesterol-modulated spatial distribution of spike^WT1268^ clusters. Scale bars, 5 μm. Scale bar applies to all images in this panel. **b.** Box plots and data points show the counts of clusters per μm^2^ of the spike^WT1268^ protein corresponding to panel **a** from N≥3 independent replicates. **c.** Box plots and data points show the size of clusters (Feret’s diameter) corresponding to panel **a** from N≥3 independent replicates. Cluster quantification and diameter measurements were performed using NIS Elements software, with ≥20 randomly selected cells in each group. Statistical analysis was performed using one-way ANOVA followed by Tukey’s multiple comparisons test.

Figure. S15.

**
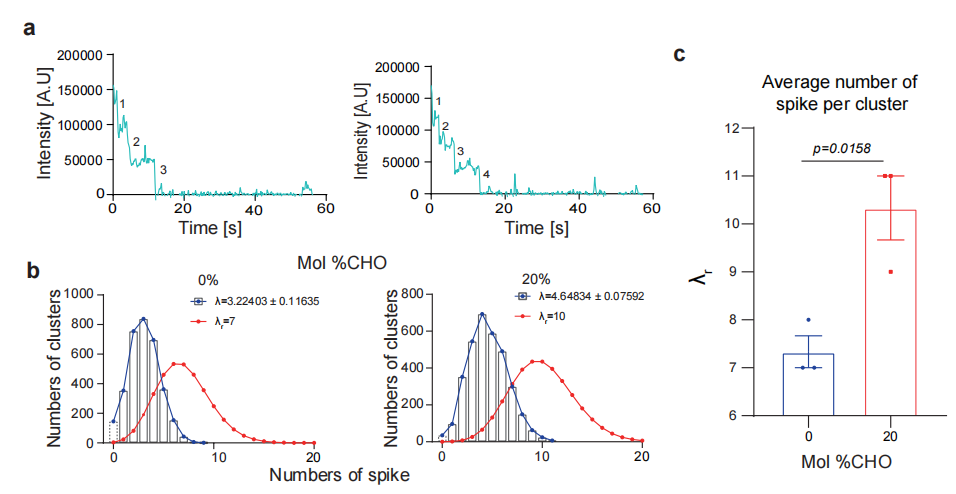
**

**Fig. S15. Single-molecule photobleaching analysis reveals cholesterol-dependent oligomerization of spike^1268^. a.** Representative time traces showing single-molecule stepwise photobleaching events with the fluorophore Cy5. **b.** Distribution of the number of spike^1268^ monomers per cluster on SLBs with 0 mol% or 20 mol% cholesterol. Blue curves: labeled monomers (λ); red curves: total monomers (λ_r_, labeled + unlabeled). **c.** Bar graphs and data points show the average number (λ_r_) of spike^1268^ per cluster on SLBs, which is obtained from the calculated Poisson distribution of all bound spike^1268^ monomers (i.e., red curves in **b**). Statistical analysis was performed using t-test. CHO, cholesterol. Statistical significance in panel **c** was assessed by unpaired t-test.

Figure. S16.


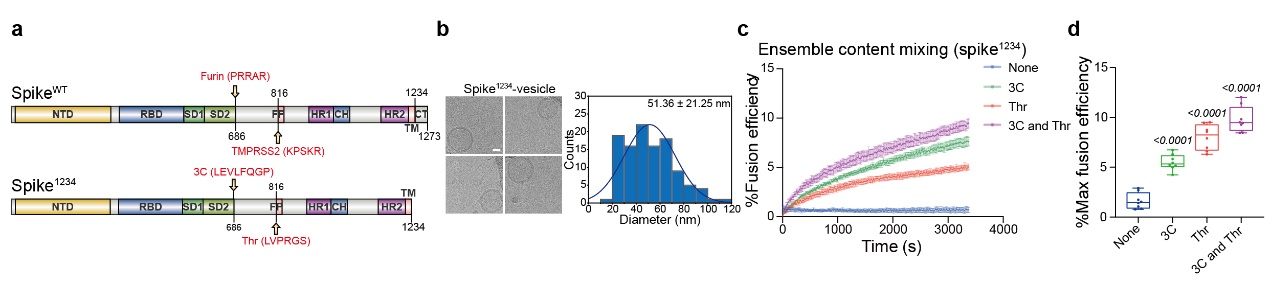


**Fig. S16. Functional reconstitution of spike^1234^-mediated ensemble content-mixing assay. a.** Domain schematic of spike^1234^ in which functional domains and cleavage sites are annotated. 39 amino acids from the C-terminal cytoplasmic domain were deleted to obtain spike^1234^. **b.** Cryo-EM images of spike^1234^-vesicle with corresponding diameter distribution histogram. Scale bars, 20 nm. Scale bar applies to all images in this panel. **c.** The ensemble content mixing assay of the spike^1234^-vesicle with ACE2-vesicle. The fluorescence change of SRB was normalized with respect to the maximum fluorescence intensity obtained by adding 0.1% Triton X-100. Traces represent the mean ± SEM from N=8 independent replicates. **d.** Box plots and data points show the maximum fusion efficiency corresponding to panel **c** Statistical analysis was performed using one-way ANOVA followed by Tukey’s multiple comparisons test.

Figure. S17.


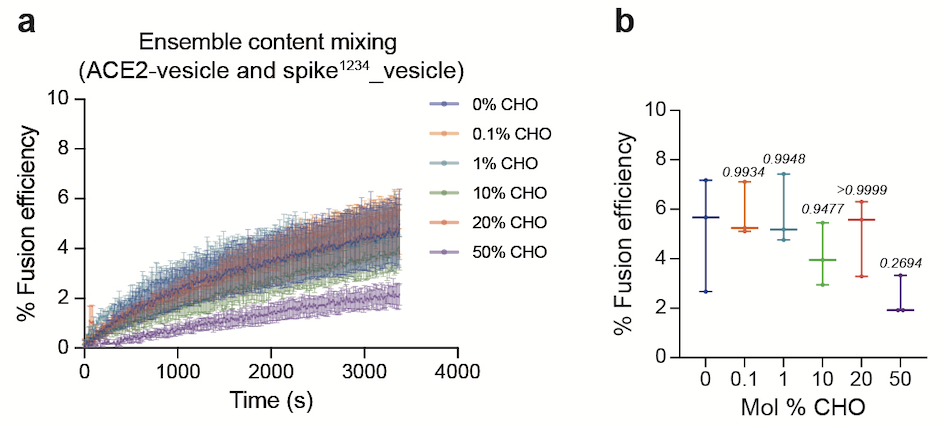


**Fig. S17.** **The ensemble content mixing of the titration of cholesterol in ACE2-vesicle with spike^1234^-vesicle. a.** The ensemble content mixing of the spike^1234^-vesicle (20 mol%) and the ACE2-vesicle (0-50 mol%). Traces represent the mean ± SEM from N=3 independent experiments. **b.** Box plots and data points show the maximum fusion efficiency corresponding to panel **a**. CHO, cholesterol. Statistical analysis was performed using one-way ANOVA followed by Tukey’s multiple comparisons test.

Figure. S18.


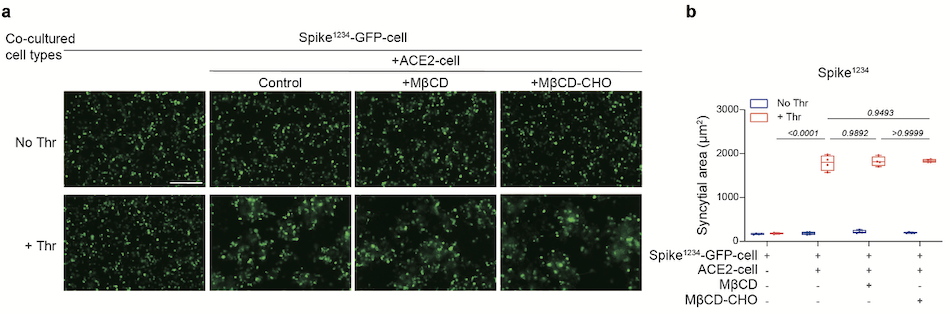


**Fig. S18.** **Cholesterol has no effect on spike^1234^-mediated syncytium formation. a.** Syncytia formation induced by spike^1234^-mediated cell-cell fusion. Scale bar, 200 μm. Scale bar applies to all images in this panel. **b.** Box plots and data points show the syncytial area in cell-cell fusion corresponding to panel **a** from N=4 independent replicates. Statistical analysis was performed using two-way ANOVA followed by Tukey’s multiple comparisons test.

Figure. S19.


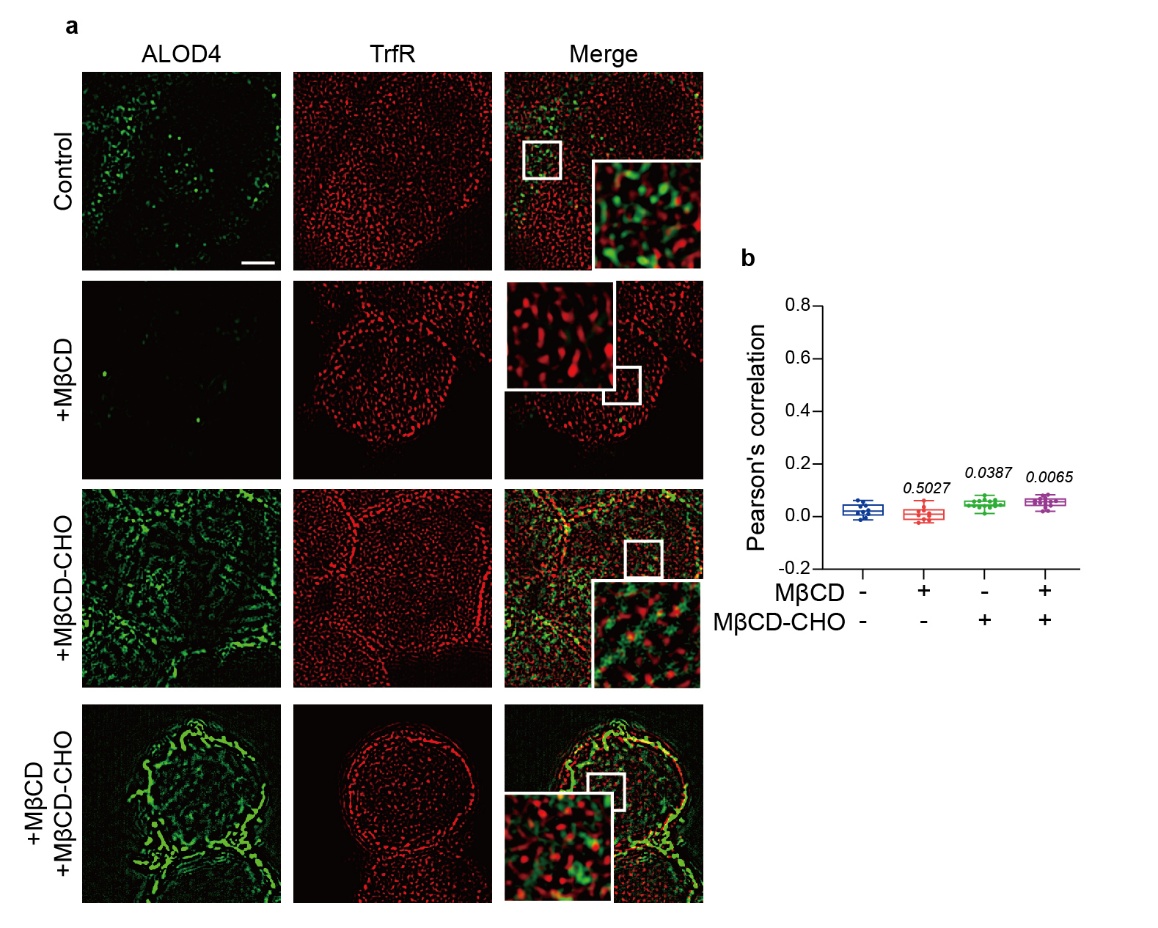


**Fig. S19. TrfR serves as a negative control showing no colocalization with cholesterol. a.** The representative fields of spatial co-localization of TrfR protein (red) and cholesterol (green) on the plasma membrane. Scale bars, 5 μm. Scale bar applies to all images in this panel. **b.** Quantification of the spatial colocalization of TrfR protein (red) and cholesterol (green) by PCC analysis, across four treatment groups (Control, +MβCD, +MβCD-CHO, +MβCD + MβCD-CHO). Statistical analysis was performed using two-way ANOVA followed by Tukey’s multiple comparisons test.

Figure. S20.


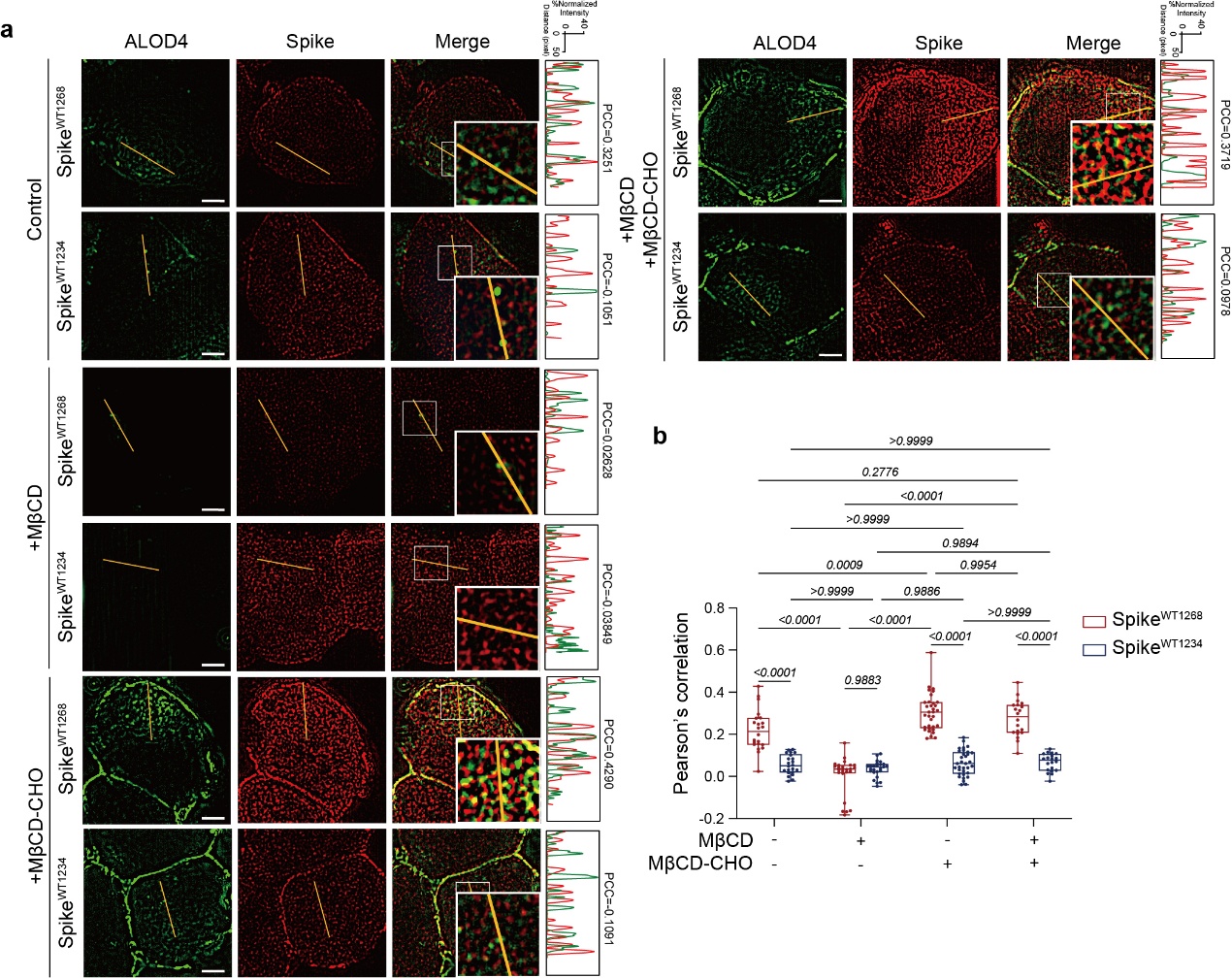


**Fig. S20. Cholesterol-driven cluster formation with spike^WT1268^ contrasts with non-colocalizing spike^WT1234^ variant. a.** The representative fields of spatial co-localization of spike^WT1268^/spike^WT134^ protein (red) and cholesterol (green) on the plasma membrane. Scale bars, 5 μm. Scale bar applies to all images in this panel. Corresponding fluorescence intensity profiles of spike^WT1268^/spike^WT1234^ protein (red) and cholesterol (green) along linear membrane regions in treated cell membrane are present on the right of the fluorescence images. **b.** Box plots and data points show comparative analysis of Pearson Correlation Coefficients (PCC) for colocalization between spike variants (spike^WT1268^ and spike^WT1234^, red) and cholesterol (green), across four cell membrane treatment groups corresponding to panel **a** from N≥4 independent replicates. ROI=22 (control), 25 (+MβCD), 33 (+MβCD-CHO), 20 (+MβCD +MβCD-CHO). In panel **b**, statistical analysis was performed using one-way ANOVA followed by Tukey’s multiple comparisons test.

Figure. S21.


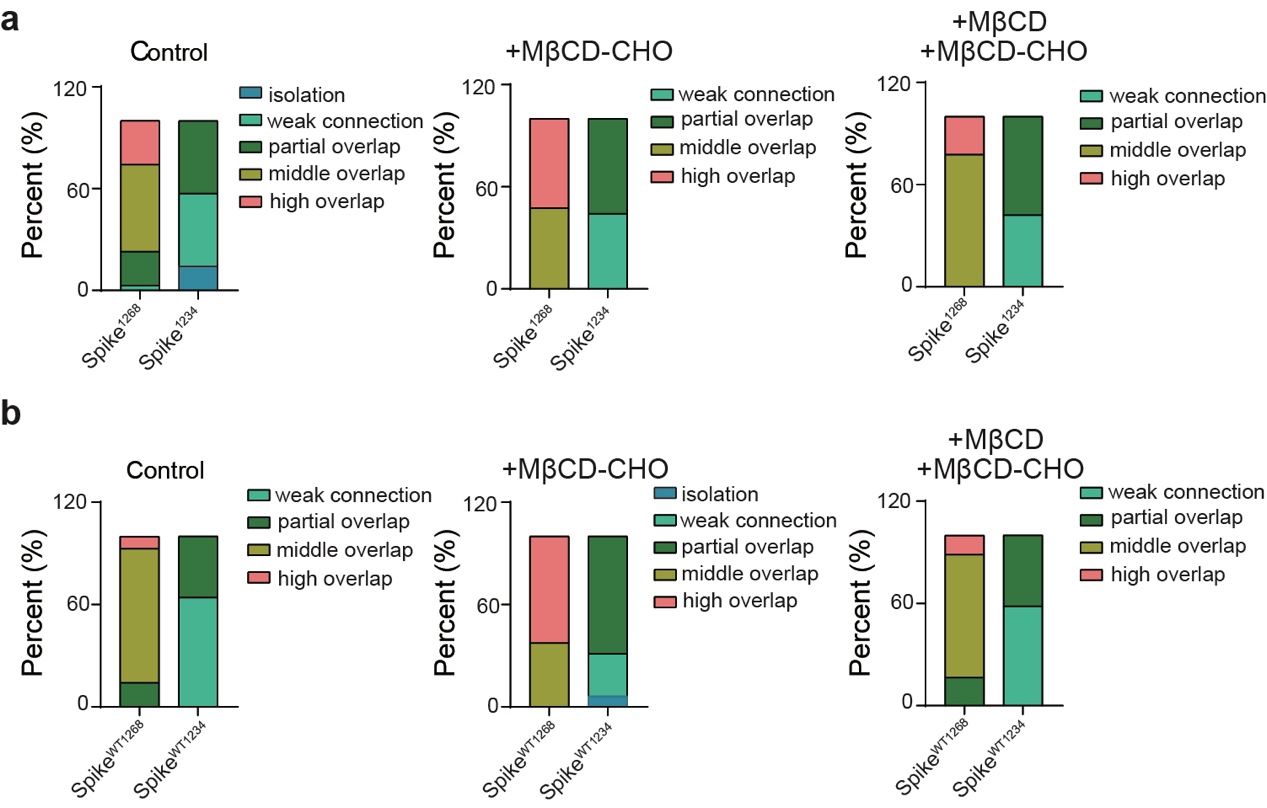


**Fig. S21.** Colocalization analysis of spike with cholesterol using Costes method. **a.** Comparative colocalization analysis between two spike variants (spike^1268^ and spike^1234^) and cholesterol. **b.** Independent colocalization analysis of individual spike variants (spike^WT1268^, left; spike^WT1234^, right) with cholesterol. We encountered NAN values when analyzing the MβCD-treated group. This occurred because the cholesterol signal in this group was extremely low, falling below the detectable threshold and resulting in NAN outputs during quantification.

Figure. S22.

**
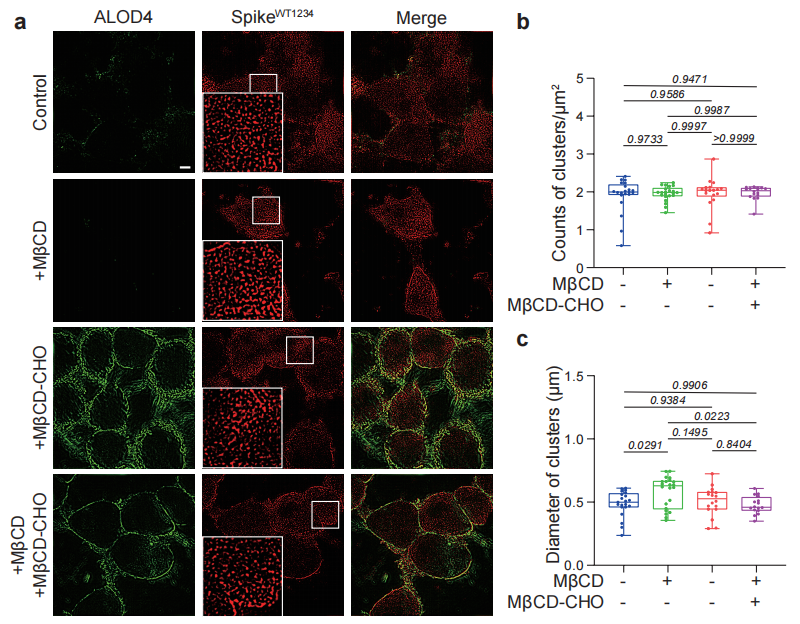
**

**Fig. S22.** **Cholesterol does not alter spatial distribution of spike^WT1234^.** **a.** Reconstructed SIM images illustrating cholesterol-modulated spatial distribution of spike^WT1234^ clusters. Scale bars, 5 μm. Scale bar applies to all images in this panel. **b.** Box plots and data points show the counts of clusters per μm^2^ for the spike^WT1234^ protein corresponding to panel **a** from N=4 independent replicates. **c.** Box plots and data points show the size of clusters (Feret’s diameter) corresponding to panel **a** from N=4 independent replicates. Cluster quantification and diameter measurements were performed using NIS Elements software, with ≥16 randomly selected cells in each group. In panels **b** and **c**, Statistical analysis was performed using one-way ANOVA followed by Tukey’s multiple comparisons test.

Figure. S23.

**
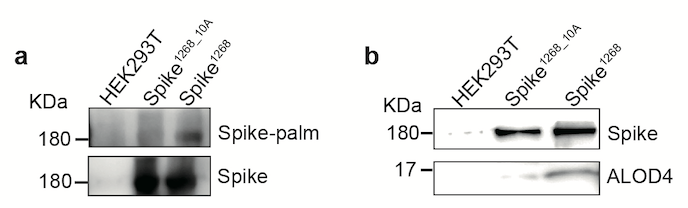
**

**Fig. S23. Cysteines mediate spike^1268^ palmitoylation and cholesterol binding. a.** Acyl-biotin exchange assay showing palmitoylation in spike^1268^ and spike^1268_10A^ protein (HEK293T lysate negative control). The palmitoylation modification was detected by HRP-streptavidin (upper), and the spike protein was detected by 1A9 antibody (lower). **b.** ALOD4-based cholesterol pull-down assay showing cysteine-dependent lipid binding. The spike protein was detected by 1A9 antibody (upper), and cholesterol was detected by ALOD4 (lower).

Figure. S24.

**
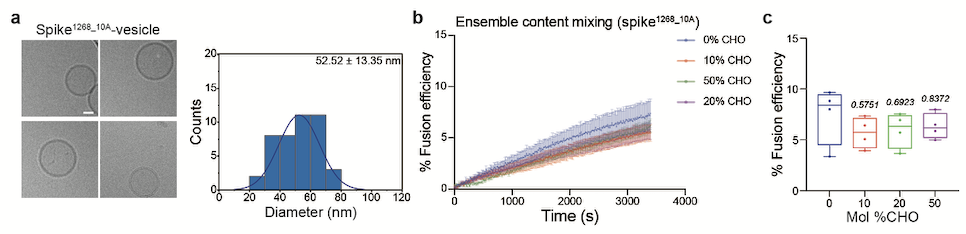
**

**Fig. S24. The ensemble content mixing of ACE2-vesicle and spike^1268_10A^-vesicle containing different concentrations of cholesterol. a.** Cryo-EM images of spike^1268_10A^-vesicle, with corresponding diameter distribution histogram. **b.** The ensemble content mixing of the spike^1268_10A^-vesicle (0-50 mol% cholesterol) and the ACE2-vesicle (20 mol% cholesterol). Traces represent the mean ± SEM from N=4 independent experiments. **c.** Box plots and data points show the maximum fusion efficiency corresponding to panel. CHO, cholesterol. In panel **c**, Statistical analysis was performed using one-way ANOVA followed by Tukey’s multiple comparisons test.

Figure. S25.

**
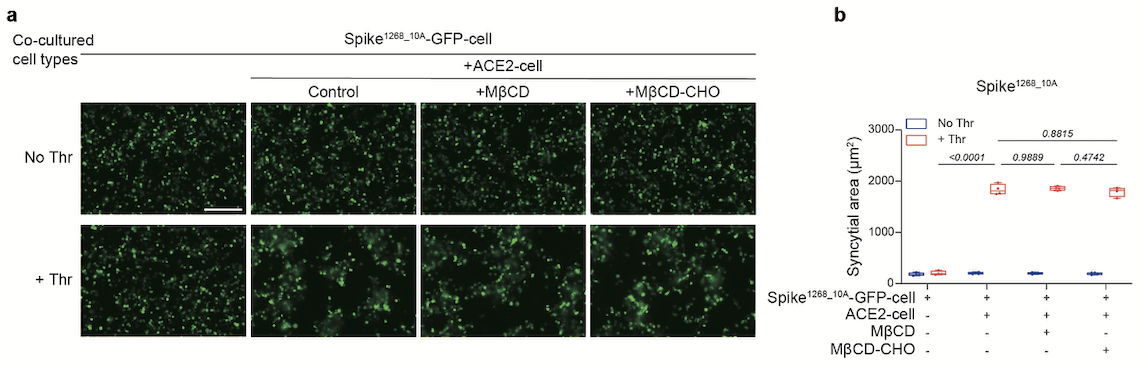
**

**Fig. S25. Cholesterol has no effect on spike^1268_10A^-mediated syncytium formation. a.** Syncytia formation induced by spike^1268_10A^-mediated cell-cell fusion. Scale bar, 200 μm. Scale bar applies to all images in this panel. **b.** Box plots and data points show the syncytial area in cell-cell fusion corresponding to panel **a** from N=4 independent replicates. Statistical analysis was performed using two-way ANOVA followed by Tukey’s multiple comparisons test.

Figure. S26.


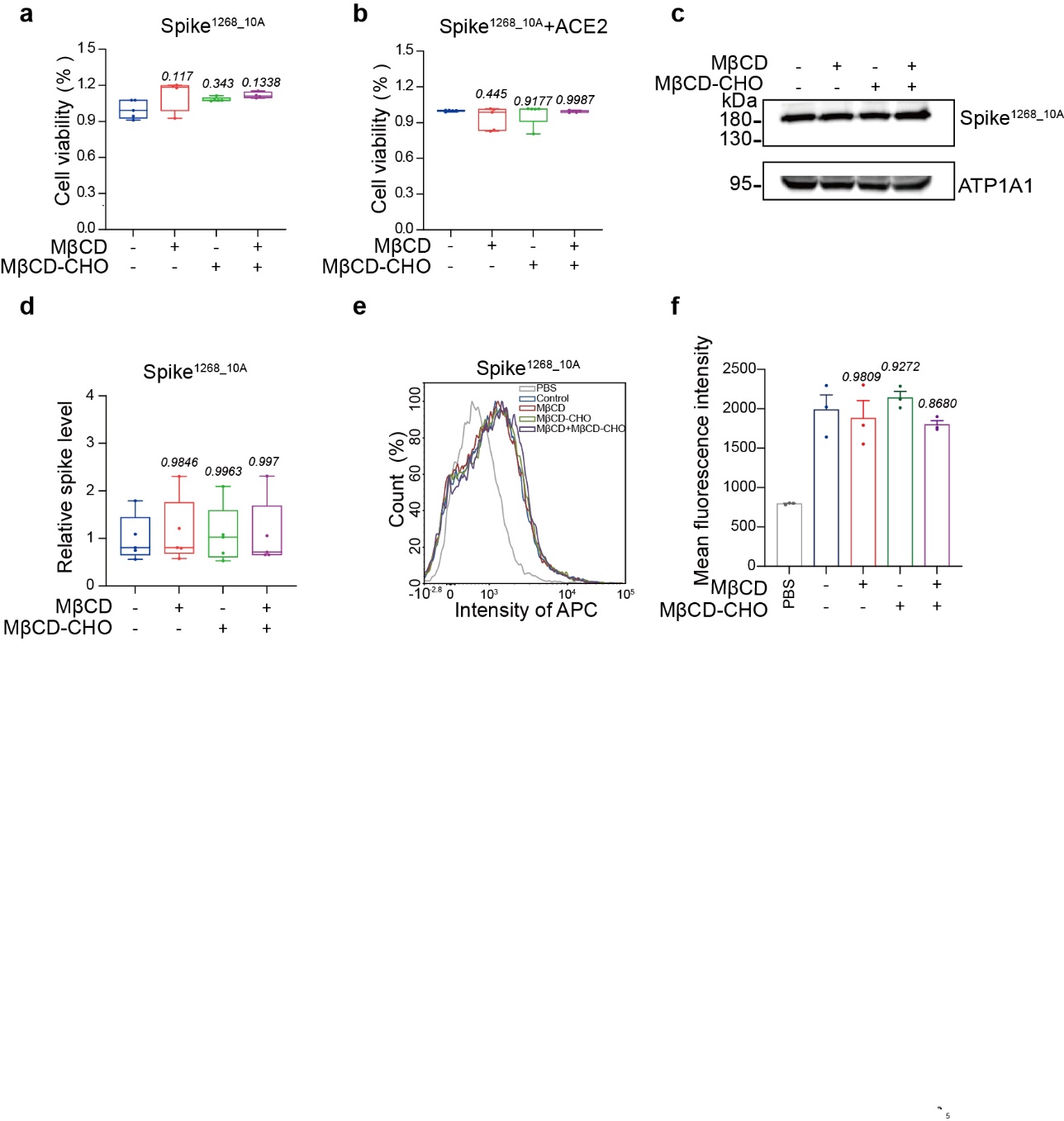


**Fig. S26. Quantification of membrane spike^1268_10A^ protein levels and assessment of cell viability with different treatments.** **a** CCK-8 assays assessing cell viability following corresponding treatments for spike^1268_10A^-GFP-cell. **b** CCK-8 measurements after 48-hour coculture with ACE2 under the same treatment conditions for spike^1268_10A^-GFP-cells**. c** Immunoblots of spike^1268_10A^ on membrane fraction following corresponding treatments. **d** Box plots and data points show the expression of spike^1268_10A^ on cell membrane corresponding to panel **c** from N=5 independent replicates. **e** Flow cytometry analysis of spike protein expression on cell membrane of spike^1268_10A^ following corresponding treatments. **f** Quantification of spike^1268_10A^ with fluorescence intensity values averaged from N=3 independent replicates. In panels **a, b, d, f**, statistical analysis was performed using one-way ANOVA followed by Tukey’s multiple comparisons test.

Figure. S27.


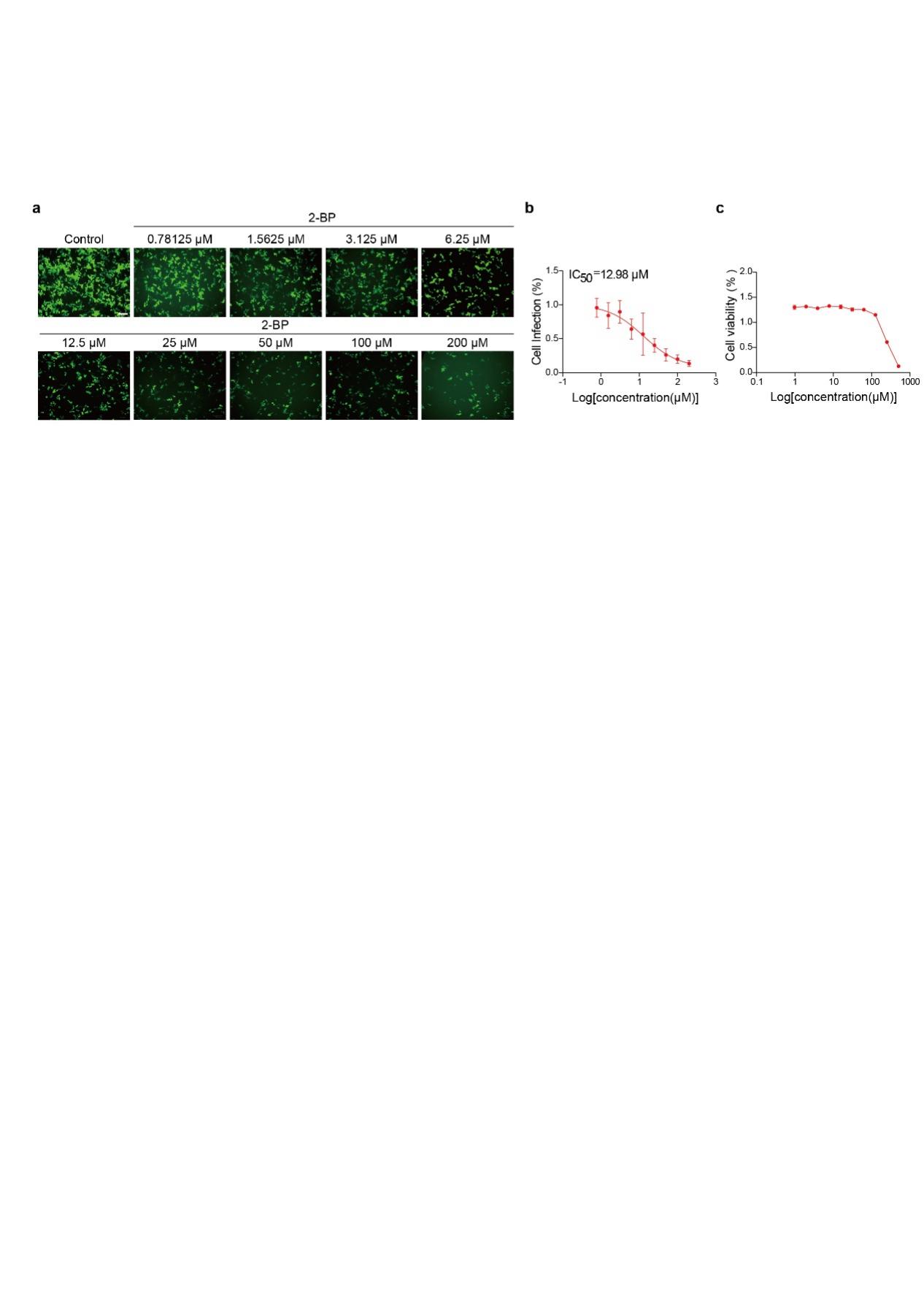


**Fig. S27. Palmitoylation inhibition impairs the infection by SARS-CoV-2 pseudovirus.** **a.** The representative fields of SARS-CoV-2 pseudovirus infection in 293-ACE2 cells with 0-200 μM 2-BP pretreatment. Scale bars, 100 μm. Scale bar applies to all images in this panel. **b.** Dose-dependent inhibition of pseudovirus infection by cholesterol replenishment. SARS-CoV-2 pseudovirus was pre-treated with increasing concentrations of 2-BP (0-200 μM). Infection efficiency was quantified and normalized, showing a cholesterol-dependent inhibition of infectivity with an IC_50_ of 12.98 μM. **c.** CCK-8 assays assessing cell viability for ACE2 expressing 293T cells with 0-500 μM 2-BP pretreatment.

Figure. S28.


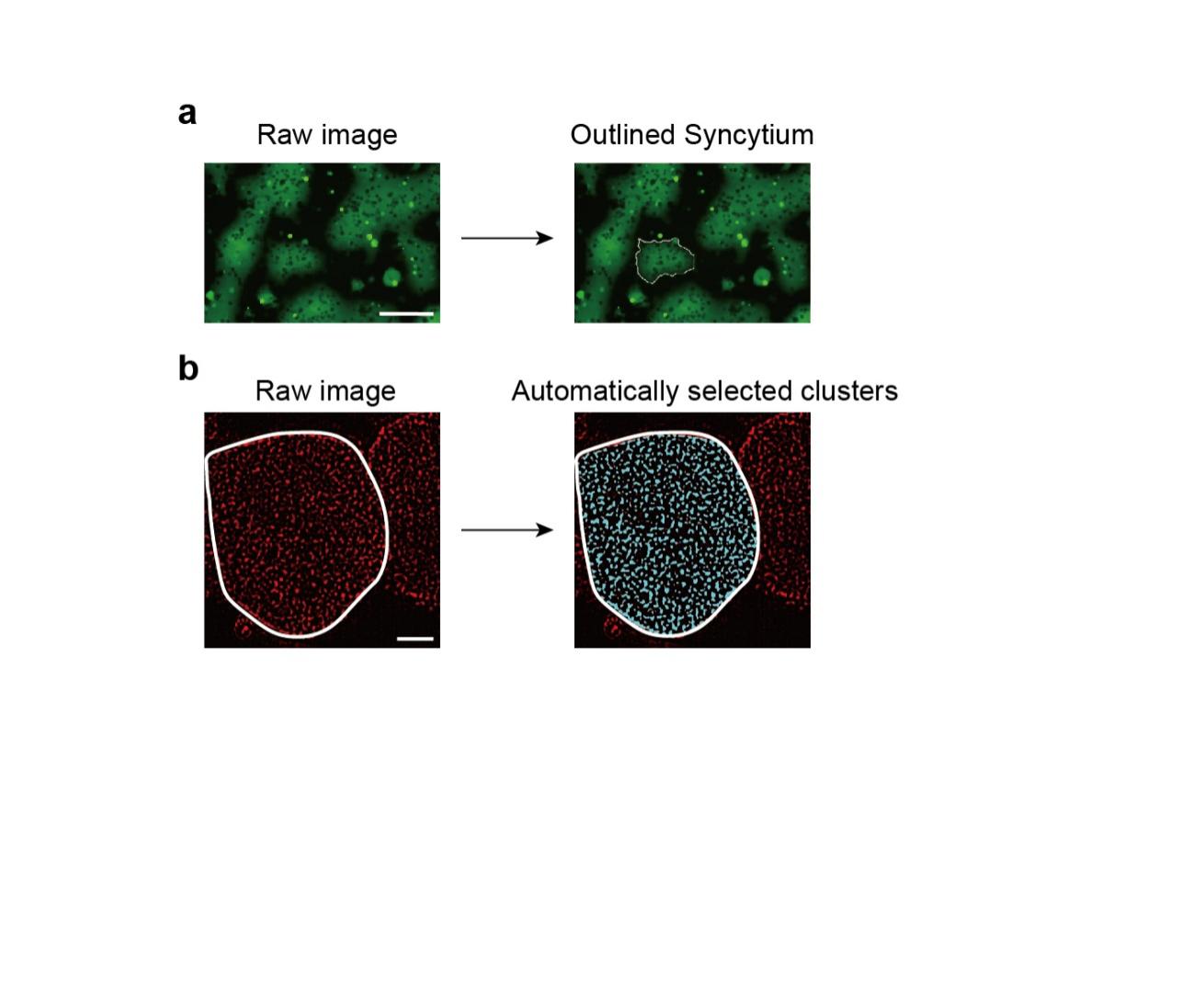


**Fig. S28. Quantitative image analysis for microscopy-based methods. a.** The measurement of syncytium. The raw image is adapted from the control group of Fig. S10b. **b.** The automated selection of the cluster of spike proteins for image analysis. The raw image is adapted from the control group of Fig.6g.
